# Different doses of tenecteplase vs. alteplase for acute ischemic stroke within 4.5 hours of symptom onset: a network meta-analysis of randomized controlled trials

**DOI:** 10.3389/fneur.2023.1176540

**Published:** 2023-06-02

**Authors:** Huo Liang, Xue Wang, Xuemei Quan, Shijian Chen, Bin Qin, Shuolin Liang, Qiuhui Huang, Jian Zhang, Zhijian Liang

**Affiliations:** ^1^Department of Neurology, The First Affiliated Hospital of Guangxi Medical University, Nanning, China; ^2^Department of Neurology, The People's Hospital of Guangxi Zhuang Autonomous Region, Nanning, China

**Keywords:** tenecteplase, alteplase, ischemic stroke, acute stroke, network meta-analysis

## Abstract

**Background:**

The optimal dose of tenecteplase vs. alteplase for acute ischemic stroke (AIS) has yet to be established. Therefore, we included the latest randomized controlled trials (RCT) to assess the efficacy and safety of different doses of tenecteplase vs. alteplase for AIS within 4.5 hours of symptom onset.

**Methods:**

Literature was searched in PubMed, Cochrane Library, Embase, Web of Science, and clinical trial registries until February 12, 2023. Odds ratios (OR) with 95% credible intervals (CrI) were estimated using Bayesian network meta-analysis (NMA). Treatments were ranked based on efficacy and safety using the surface under the cumulative ranking curve (SUCRA).

**Results:**

Eleven RCTs with 5,475 patients were included. Tenecteplase 0.25 mg/kg and alteplase 0.9 mg/kg had significantly higher rates of excellent functional outcome (tenecteplase: OR, 1.85; 95% CrI, 1.44–2.37; alteplase: OR, 1.60; 95% CrI, 1.29–1.97) and good functional outcome (tenecteplase: OR, 1.54; 95% CrI, 1.19–1.98; alteplase: OR, 1.40; 95% CrI, 1.14–1.74) than placebo, despite an increased risk of symptomatic intracranial hemorrhage. Furthermore, the NMA (OR, 1.16; 95% CrI, 1.01–1.33) and the pairwise meta-analysis (OR, 1.16; 95% CI, 1.02–1.33; P = 0.03) indicated that tenecteplase 0.25 mg/kg was superior to alteplase 0.9 mg/kg in excellent functional outcome. Alteplase 0.9 mg/kg (OR, 2.54; 95% CrI, 1.45–8.08) significantly increased the risk of any intracranial hemorrhage compared with placebo. SUCRA results demonstrated that tenecteplase 0.25 mg/kg ranked first and tenecteplase 0.4 mg/kg ranked last in efficacy outcomes.

**Conclusions:**

The NMA indicated that tenecteplase 0.25 mg/kg and alteplase 0.9 mg/kg are safe and significantly improve clinical outcomes in patients with AIS within 4.5 h of symptom onset. Furthermore, tenecteplase 0.25 mg/kg provides more benefit and has the potential to replace alteplase 0.9 mg/kg in AIS treatment.

**Systematic review registration:**

https://www.crd.york.ac.uk/PROSPERO/index.php, identifier: CRD42022343948.

## Introduction

Stroke is the leading cause of disability and the second largest cause of death worldwide (1). Despite international guidelines recommending tenecteplase as an alternative in specific subgroups of acute ischemic stroke (AIS), alteplase remains the only intravenous thrombolytic agent approved by the United States Food and Drug Administration for treating AIS (2, 3). However, alteplase has several drawbacks, such as having a short half-life and requiring continuous infusion for approximately an hour. Tenecteplase is a genetic variant of alteplase with better pharmacological characteristics, including a higher specificity for fibrinogen, stronger resistance to plasminogen activator inhibitor-1, a prolonged half-life, rapid thrombolysis, and less fibrinogen depletion. These modifications make it more efficient for thrombolysis and easier to administer rapidly with a single intravenous bolus without requiring equipment such as infusion pumps. Several clinical trials suggested the benefit of tenecteplase vs. alteplase, but the results are still inconclusive (4, 5).

Traditional meta-analyses make it difficult to assess the effects of two or more interventions that are not directly comparable. In contrast, network meta-analysis (NMA) can provide indirect comparative evidence by comparing two or more treatments that have never been directly compared through a common comparator (6, 7). NMA can provide higher statistical precision by integrating direct and indirect evidence, which pairwise meta-analyses do not consider (6, 7). Furthermore, the NMA can provide valuable information on superior treatment through ranking analysis. The information helps select treatment options and develop guidelines (6).

Current high-quality evidence suggests that intravenous thrombolysis (IVT) with alteplase within 4.5 h of symptom onset improves the clinical outcomes of AIS patients. Beyond 4.5 h, the risk might outweigh the benefit (8, 9). Several NMAs on the topic had previously been published. However, to our knowledge, these meta-analyses included patients with AIS beyond and within 4.5 h of symptom onset. A mixed analysis of data from both time windows beyond and within 4.5 h may not accurately convey the strength of the evidence for the core indicator of thrombolysis within 4.5 h. In addition, a recent large randomized controlled trial (RCT) from China showed that tenecteplase was non-inferior to alteplase in patients with AIS within 4.5 h of symptom onset (10). We believe this study will provide more comprehensive evidence to assess the efficacy and safety of tenecteplase and alteplase. Therefore, we incorporated the latest RCTs and performed a systematic review and NMA to compare the effectiveness and safety of various doses of tenecteplase vs. alteplase for AIS within 4.5 h of symptom onset.

## Methods

Following the Preferred Reporting Items for Systematic Reviews and Meta-Analyses (PRISMA) extension statement for reporting NMA, this systematic review and NMA was performed based on a prospective registration (PROSPERO CRD42022343948).

### Literature search

The literature searches in PubMed, Embase, Web of Science, Cochrane library, and clinical trial registries were performed independently by three researchers (HL, QH, and SL). Our search was unrestricted by language, year, or publication status. The last literature search was performed on February 12, 2023. Supplementary Table 1 details the search algorithm.

### Inclusion criteria

RCTs were incorporated, which assessed various doses of tenecteplase and standard-dose (0.9 mg/kg) alteplase for AIS within 4.5 h of symptom onset. Patients in the intervention were treated with various doses of tenecteplase or standard dose alteplase, and the comparison group was treated with standard dose alteplase or placebo. At least one efficacy or safety outcome must be reported in the included literature. The NMA excluded basic experimental studies, conference abstracts, case reports, reviews, and studies with overlapping participant data.

### Data extraction and outcome measures

Two reviewers (XW and BQ) independently extracted study features (first author's name or study name, publication date, study design, country, recruitment time, treatment time window, type of intervention, number of patients in each treatment arm, age, gender ratio, baseline National Institutes of Health Stroke Scale (NIHSS) score, time from onset to treatment), efficacy, and safety outcomes from each eligible study. The efficacy outcomes included excellent functional outcome, defined as a modified Rankin scale (mRS) score of 0–1 at 3 months, and good functional outcome (defined as mRS 0–2 at 3 months). The safety outcomes included mortality at 3 months, symptomatic ICH (sICH), and any ICH. A type of ICH discovered on follow-up CT post-thrombolysis was considered any ICH. Controversies were resolved by consensus.

### Risk of bias assessment

The Cochrane Risk Bias Assessment Tool was used independently by two reviewers (XQ and SC) to assess the quality of the included RCT in seven domains, including random sequence generation, allocation concealment, blinding of participants and personnel, blinding of outcome assessment, selective reporting, incomplete outcome data, and other bias (11). Arguments were settled by consensus. A risk of bias plot was created using Review Manager (Version 5.4).

### Statistical analysis

Transitivity is a crucial assumption for an NMA. Before performing the NMA, the clinical and methodological characteristics of the included studies were thoroughly evaluated to determine whether the transitivity assumption was valid. Studies with a sufficiently comparable distribution of effect modifiers qualified for data synthesis. A random effects inconsistency model was fitted. The deviance information criteria (DIC) and the posterior mean deviance of each data point were compared with those from the corresponding consistency model to evaluate the global inconsistency of direct and indirect evidence in the network (12). Additionally, the node-splitting models were employed to evaluate the local inconsistency between direct and indirect comparisons (13). The heterogeneity between the studies was assessed using the global I-squared (I^2^) statistic in this NMA. A value of >50% indicated significant heterogeneity between the studies (14).

A Bayesian NMA with the non-informative prior distributions and pairwise meta-analysis were performed in R (Version 4.2.1) and RStudio (Version 2022.07.0 Build 548) using the packages “gemtc”, “rjags”, “ggplot2”, “meta”, and “BUGSnet (Version 1.1.1) (15)”. Model fit was evaluated using leverage graphs, displaying the corresponding effective number of parameters (pD), posterior mean of the residual deviance (Dres), and DIC (15, 16). A model was chosen based on the lower DIC value, indicating a better fit, and a difference of more than five points, indicating a significant difference (12). The Markov Chain Monte Carlo algorithm was used for each outcome with a burn-in of 10 000 iterations followed by 100 000 with 5,000 adaptations. Trace plots and Gelman-Rubin diagnostics were used to evaluate model convergence (Supplementary Figures 1–5) (15, 17). Network plots were created to demonstrate which treatments were compared directly within the RCT. The pooled results were provided as odds ratios (OR) with 95% credible intervals (95% CrI). Additionally, the surface under the cumulative ranking curve (SUCRA) was used to compute the probability of ranking each treatment effect for each intervention (18). The R statistical package was used for data analysis. A statistically significant difference was established at *P* < 0.05.

## Results

### Search results and characteristics of included trials

Our literature search revealed 6,336 papers, and after eliminating duplicates and screening titles and abstracts, we ultimately evaluated 29 full-text articles. After screening and selection, 11 RCTs met the criteria for inclusion in the NMA (4, 10, 19–27). We utilized a flowchart to summarize the screening results (Supplementary Figure 6). Two papers reported ATLANTIS Trial results, and we extracted data from one of them for treating AIS within 3 h of symptom onset (23, 28). A total of 5,475 patients were randomized, with 1,818 receiving tenecteplase 0.25 mg/kg, 91 receiving tenecteplase 0.1 mg/kg, 60 receiving tenecteplase 0.32 mg/kg, 119 receiving tenecteplase 0.4 mg/kg, 2,634 receiving alteplase 0.9 mg/kg, and 753 receiving placebo. The primary research characteristics of the 11 RCTs were summarized in Table 1. Supplementary Figure 7 presents the network plots of eligible comparisons for efficacy and safety outcomes. Supplementary Figure 8 summarizes the risk of bias in the included studies. Bias was mainly attributable to the lack of blinding of participants and personnel in eight RCTs judged to be at a “high” risk of bias.

**Table 1:** Characteristics of included studies.

**Study**	**Year**	**Study design**	**Country**	**Recruitment time**	**Time window**	**Type of intervention and doses (mg/kg)**	**No. of patients**	Age, years^*^	**Male sex (%)**	**Baseline NIHSS** score^*^	Time from onset to treatment (min) ^*^
NINDS	1995	RCT	United States	1991–1994	3 h	ALT 0.9	Part 1: 144; Part 2: 168	Part 1: 67 ± 10; Part 2: 69 ± 12	Part 1: 58; Part 2: 57	Part 1: median = 14; Part 2: median = 14	NA
						Placebo	Part 1: 147; Part 2: 165	Part 1: 66 ± 11; Part 2: 66 ± 13	Part 1: 60; Part 2: 58	Part 1: median = 14; Part 2: median = 15	NA
ATLANTIS	1999	RCT	United States	1993–1998	3 h	ALT 0.9	23	66 ± 10.4	82.6	12 ± 7.6	161 ± 21
						Placebo	38	66 ± 10.7	57.9	12 ± 4.9	144 ± 33
ECASS III	2008	RCT	European countries	2003–2007	4.5 h	ALT 0.9	418	64.9 ± 12.2	63.2	10.7 ± 5.6	median = 239
						Placebo	403	65.6 ± 11.0	57.3	11.6 ± 5.9	median = 238
Haley	2010	RCT	United States	2006–2008	3 h	TNK 0.1	31	67 ± 19	39	8 (5–11)	NA
						TNK 0.25	31	69 ± 15	52	10 (6–15)	
						TNK 0.4	19	68 ± 16	68	9 (5–17)	
						ALT 0.9	31	72 ± 16	51	13 (5–17)	
ATTEST	2015	RCT	Scotland	2012–2013	4.5 h	TNK 0.25	52	71 ± 13	64	12 (9–18)	184 ± 44
						ALT 0.9	52	71 ± 12	63	11 (8–16)	192 ± 45
Campbell	2018	RCT	Australia and New Zealand	2015–2017	4.5 h	TNK 0.25	101	70.4 ± 15.1	57	17 (12–22)	125 (102–156)
						ALT 0.9	101	71.9 ± 13.7	51	17 (12–22)	134 (104–176)
TRACE	2022	RCT	China	2018–2020	3 h	TNK 0.1	60	62.4 ± 11.1	80	7 (5–10)	154 (56–195)
						TNK 0.25	57	64.3 ± 12.8	73.3	8 (5–12)	149 (80–179)
						TNK 0.32	60	64.8 ± 12.1	70	7.5 (6–12)	147 (69–220)
						ALT 0.9	59	66.5 ± 12.6	64.4	8 (5–12)	153 (18–187)
TASTE-A	2022	RCT	Australia	2019–2021	4.5 h	TNK 0.25	55	76 (60–84)	60	8 (5–14)	97 (68–157)
						ALT 0.9	49	73 (61–80)	61	8 (5–17)	92 (66–31)
NOR-TEST 2, part A	2022	RCT	Norwegian	2019–2021	4.5 h	TNK 0.4	100	73.2 ± 12.6	45	11.5 (8–17)	92.5 (74–143)
						ALT 0.9	104	68.6 ± 15.6	51	11 (8–17.5)	99 (73–143)
AcT	2022	RCT	Canada	2019–2022	4.5 h	TNK 0.25	806	74 (63–83)	52.6	9 (6–16)	128 (93–186)
						ALT 0.9	771	73 (62–83)	51.6	10 (6–17)	131 (95–188)
TRACE2	2023	RCT	China	2021–2022	4.5 h	TNK 0.25	716	67 (58–73)	69	7 (5–10)	180 (135–222)
						ALT 0.9	714	65 (58–72)	68	7 (6–10)	178.5 (135–230)

^*^Data expressed as mean ± SD or median (IQR). SD = standard deviation; IQR = interquartile range; TNK = tenecteplase; ALT = alteplase; NA = not available.

### Transitivity, inconsistency, and heterogeneity

We did not discover significant differences between the treatments regarding the average participant age and gender, the time from onset to treatment, and the baseline NIHSS score by analyzing the characteristics of the included studies (Table 1). We considered that the transitivity assumption would hold across all studies and comparisons. The node-splitting analysis of excellent functional outcome, good functional outcome, mortality at 3 months, and any ICH revealed no evidence of local inconsistency (Supplementary Figure 9). The data of sICH could not be analyzed using the node-splitting model because there were zero sICH events in the doses of 0.25, 0.1 mg/kg tenecteplase, and placebo treatment arms in three studies. Furthermore, we evaluated the network's global consistency of direct and indirect evidence using a random effects consistency model and an inconsistency model. Both models of all outcomes had very similar Dres and DIC (Supplementary Figure 10). The posterior mean deviance comparison plots revealed that the contributions of each data point to the deviance were similar, close to the equality line for both models, suggesting no evidence of global inconsistency for all outcomes in the network (Supplementary Figure 11). Except for low heterogeneity in any ICH (I^2^.pair = 16.35%, I^2^.cons = 0%) and mortality at 3 months (I^2^.pair = 28.84%, I^2^.cons = 15.04%), the global I^2^ statistic of other outcomes did not identify any heterogeneity across the studies (Supplementary Table 2).

### Bayesian network meta-analysis

We performed Bayesian random-effects models to analyze mortality at 3 months, sICH, and any ICH. Fixed-effects models were used for excellent and good functional outcomes because the fit of fixed-effects models was better (lower DIC values, Supplementary Figure 12).

#### Excellent functional outcome

**Figure 1:**
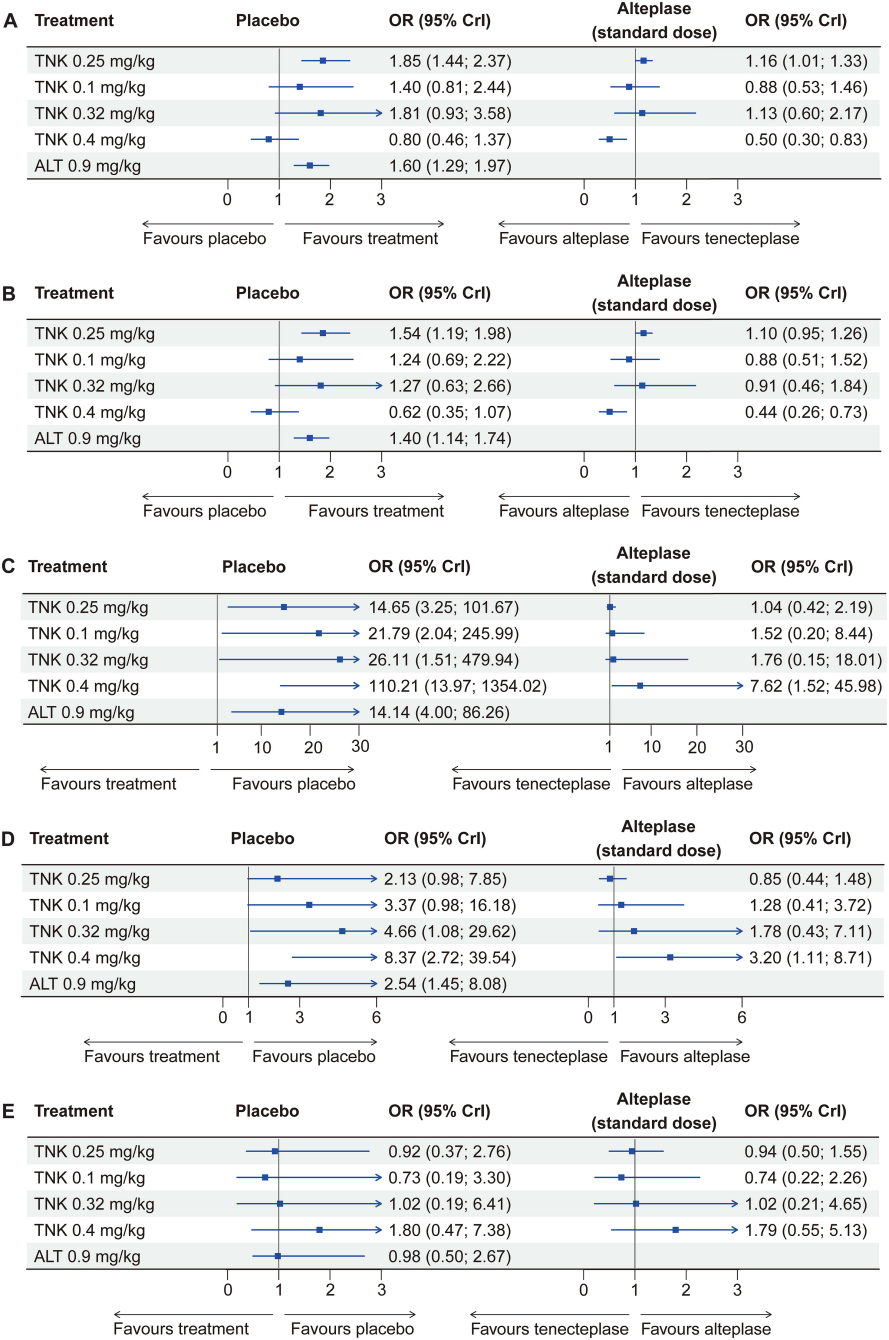
Forest plots display effect estimates of different doses of tenecteplase and standard-dose (0.9 mg/kg) alteplase compared with placebo, as well as different doses of tenecteplase compared with standard-dose alteplase for all outcomes. OR = odds ratio; CrI = credible interval; TNK = tenecteplase; ALT = alteplase. **(A)** excellent functional outcome; **(B)** good functional outcome; **(C)** symptomatic intracranial hemorrhage; **(D)** any intracranial hemorrhage; and **(E)** mortality at 3 months.

**Figure 2:**
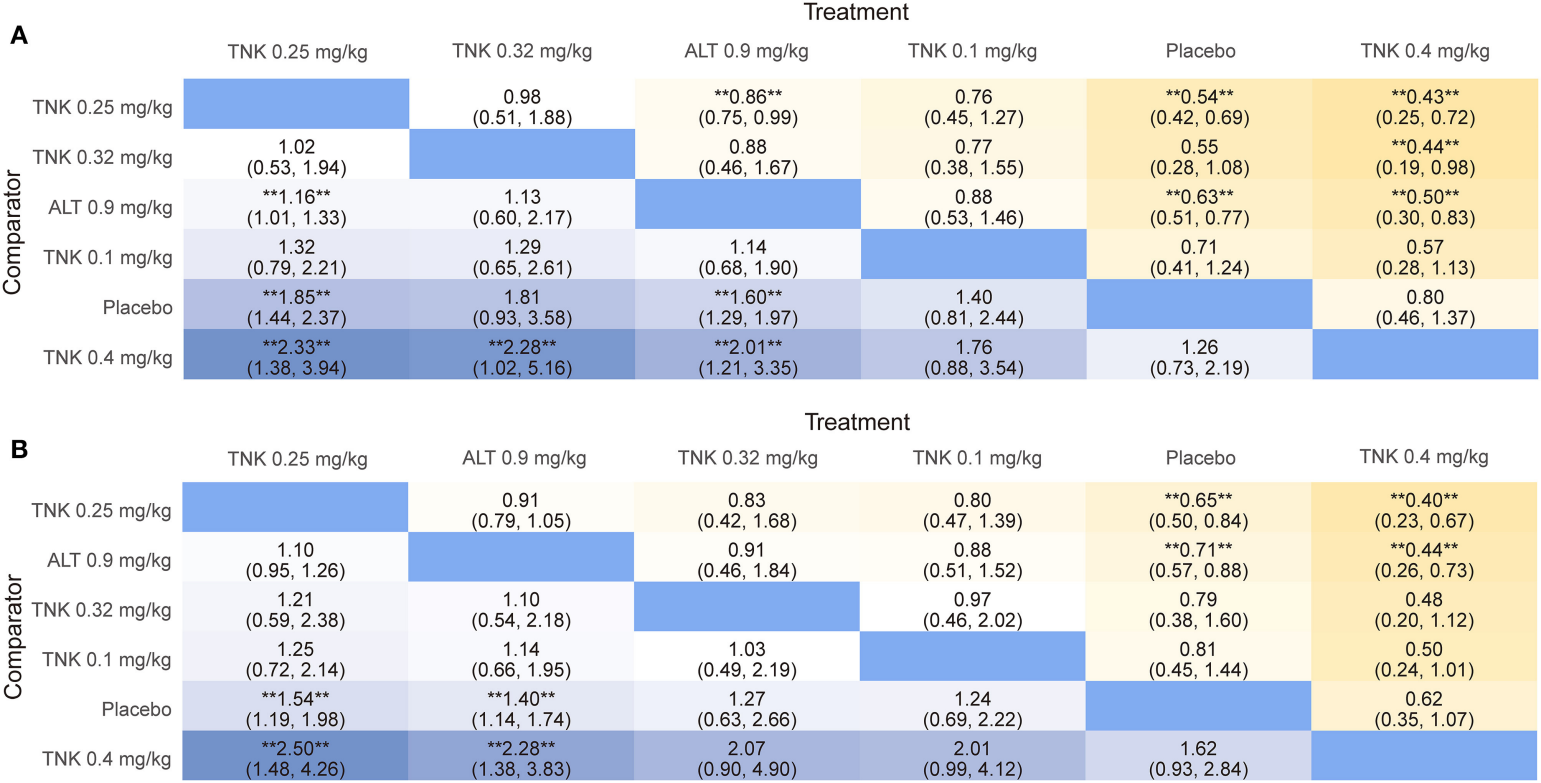
League table heatmaps for efficacy outcomes. Data are ORs (95% CrI) of the treatment on the top, compared with the comparator on the left. OR > 1.0 shows an advantage for the treatment, whereas OR <1.0 shows an advantage for the comparator. Statistically significant results (*P* < 0.05) are marked by the symbols (**). OR = odds ratio; CrI = credible interval; TNK = tenecteplase; ALT = alteplase. **(A)** excellent functional outcome; **(B)** good functional outcome.

**Figure 3:**
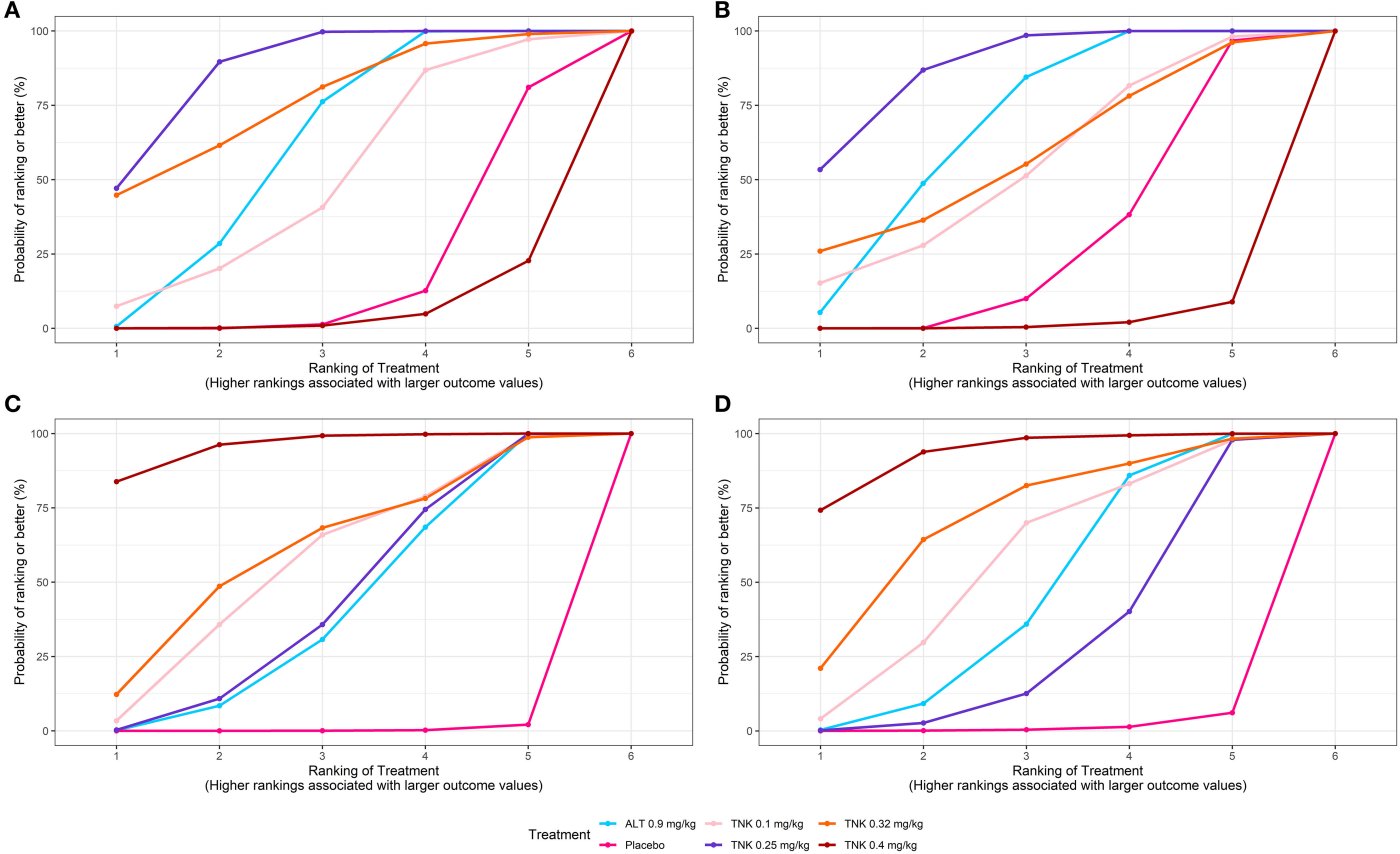
SUCRA plots for efficacy and safety outcomes. Graphs indicate the cumulative probability of each treatment ranking for each outcome. For efficacy outcomes **(A, B)**, the ranking is from best (highest ranking) to worst (lowest ranking). For safety outcomes **(C, D)**, the ranking is from worst (highest ranking) to best (lowest ranking). For example, TNK 0.25 mg/kg ranked best for improving efficacy outcomes, whereas TNK 0.4 mg/kg ranked poorest. SUCRA = surface under the cumulative ranking curve; TNK = tenecteplase; ALT = alteplase; **(A)** excellent functional outcome; **(B)** good functional outcome; **(C)** symptomatic intracranial hemorrhage; and **(D)** any intracranial hemorrhage.

Eleven RCTs, including 5,407 participants, reported 2,481 (45.88%) excellent functional outcome patients. Tenecteplase 0.25 mg/kg and alteplase 0.9 mg/kg were associated with significantly higher rates of excellent functional outcome compared with placebo (tenecteplase 0.25 mg/kg: OR, 1.85; 95% CrI, 1.44–2.37; alteplase 0.9 mg/kg: OR, 1.60; 95% CrI, 1.29–1.97) and tenecteplase 0.4 mg/kg (tenecteplase 0.25 mg/kg: OR, 2.33; 95% CrI, 1.38–3.94; alteplase 0.9 mg/kg: OR, 2.01; 95% CrI, 1.21–3.35). In addition, tenecteplase 0.32 mg/kg (OR, 2.28; 95% CrI, 1.02–5.16) had higher rates of excellent functional outcome than tenecteplase 0.4 mg/kg. Furthermore, compared to alteplase 0.9 mg/kg, tenecteplase 0.25 mg/kg exhibited significantly higher odds of excellent functional outcome (OR, 1.16; 95% CrI, 1.01–1.33). Other treatment options and placebo did not differ significantly in excellent functional outcome (Figures 1A, 2A). Figure 3A presents the SUCRA plot of excellent functional outcome. Tenecteplase 0.25 mg/kg had the highest SUCRA value at 0.87, followed by tenecteplase 0.32 mg/kg (SUCRA, 0.76), alteplase 0.9 mg/kg (SUCRA, 0.61), tenecteplase 0.1 mg/kg (SUCRA, 0.50), placebo (SUCRA, 0.19), and tenecteplase 0.4 mg/kg (SUCRA, 0.06) (Supplementary Table 3). Based on SUCRA results, tenecteplase 0.25 mg/kg was the first-ranking treatment and tenecteplase 0.4 mg/kg was the last-ranking treatment in terms of efficacy for excellent functional outcome.

#### Good functional outcome

Ten RCTs comprising 5,356 participants identified 3,251 (60.70%) people with good functional outcome. Tenecteplase 0.25 mg/kg and alteplase 0.9 mg/kg were associated with significantly higher rates of good functional outcome compared with placebo (tenecteplase 0.25 mg/kg: OR, 1.54; 95% CrI, 1.19–1.98 and alteplase 0.9 mg/kg: OR, 1.40; 95% CrI, 1.14–1.74) and with tenecteplase 0.4 mg/kg (tenecteplase 0.25 mg/kg: OR, 2.50; 95% CrI, 1.48–4.26 and alteplase 0.9 mg/kg: OR, 2.28; 95% CrI, 1.38–3.83). No statistically significant difference was observed between tenecteplase (0.1, 0.32, and 0.4 mg/kg) and placebo (Figures 1B, 2B). Figure 3B depicts the SUCRA plot of good functional outcome. Tenecteplase 0.25 mg/kg had the highest SUCRA value at 0.88, followed by alteplase 0.9 mg/kg (SUCRA, 0.68), tenecteplase 0.32 mg/kg (SUCRA, 0.58), tenecteplase 0.1 mg/kg (SUCRA, 0.55), placebo (SUCRA, 0.29), and tenecteplase 0.4 mg/kg (SUCRA, 0.02) (Supplementary Table 3). According to SUCRA values, tenecteplase 0.25 mg/kg was the first-ranking treatment and tenecteplase 0.4 mg/kg was the last-ranking treatment in terms of efficacy for good functional outcome.

#### Symptomatic ICH

Eleven RCTs with 5,447 participants revealed 139 (2.52%) sICH patients. Compared with placebo, The doses of 0.4 mg/kg (OR, 110.21; 95% CrI, 13.97–1,354.02), 0.32 mg/kg (OR, 26.11; 95% CrI, 1.51–479.94), 0.1 mg/kg (OR, 21.79; 95% CrI, 2.04–245.99), and 0.25 mg/kg (OR, 14.65; 95% CrI, 3.25–101.67) tenecteplase and 0.9 mg/kg alteplase (OR, 14.14; 95% CrI, 4.00–86.26) significantly increased the risk of sICH. In addition, tenecteplase 0.4 mg/kg significantly increased the risk of sICH compared with tenecteplase 0.25 mg/kg (OR, 7.41; 95% CrI, 1.39–48.90) and alteplase 0.9 mg/kg (OR, 7.62; 95% CrI, 1.52–45.98), and the difference was statistically significant (Figure 1C, Supplementary Figure 13A). Figure 3C displays the SUCRA plot of sICH. Tenecteplase 0.4 mg/kg had the highest SUCRA value at 0.96, followed by tenecteplase 0.32 mg/kg (SUCRA, 0.61), tenecteplase 0.1 mg/kg (SUCRA, 0.57), tenecteplase 0.25 mg/kg (SUCRA, 0.44), alteplase 0.9 mg/kg (SUCRA, 0.42), and placebo (SUCRA, <0.01). The SUCRA values (Supplementary Table 3) revealed that the risk of sICH was lowest with placebo and highest with tenecteplase 0.4 mg/kg.

### Any ICH

Eleven RCTs with 5,245 participants revealed 726 (13.84%) any ICH patients. The doses of 0.32 mg/kg (OR, 4.66; 95% CrI, 1.08–29.62), 0.4 mg/kg (OR, 8.37; 95% CrI, 2.72–39.54) tenecteplase, and 0.9 mg/kg alteplase (OR, 2.54; 95% CrI, 1.45–8.08) significantly increased the risk of any ICH compared with placebo (Figure 1D, Supplementary Figure 13B). Figure 3D illustrates the SUCRA plot of any ICH. Tenecteplase 0.4 mg/kg had the highest SUCRA value at 0.93, followed by tenecteplase 0.32 mg/kg (SUCRA, 0.71), tenecteplase 0.1 mg/kg (SUCRA, 0.57), alteplase 0.9 mg/kg (SUCRA, 0.46), tenecteplase 0.25 mg/kg (SUCRA, 0.31), and placebo (SUCRA, 0.02). The SUCRA values (Supplementary Table 3) revealed that the risk of any ICH was lowest with placebo and highest with tenecteplase 0.4 mg/kg.

#### Mortality at 3 months

Eleven RCTs with 5,424 participants revealed 620 (11.43%) mortality at 3 months. Mortality between the treatment options did not differ significantly at 3 months (Figure 1E, Supplementary Figure 13C). However, SUCRA values (Supplementary Table 3) suggested that tenecteplase 0.4 mg/kg (SUCRA, 0.84) ranked highest in the incidence of mortality at 3 months.

### Pairwise meta-analysis

**Figure 4:**
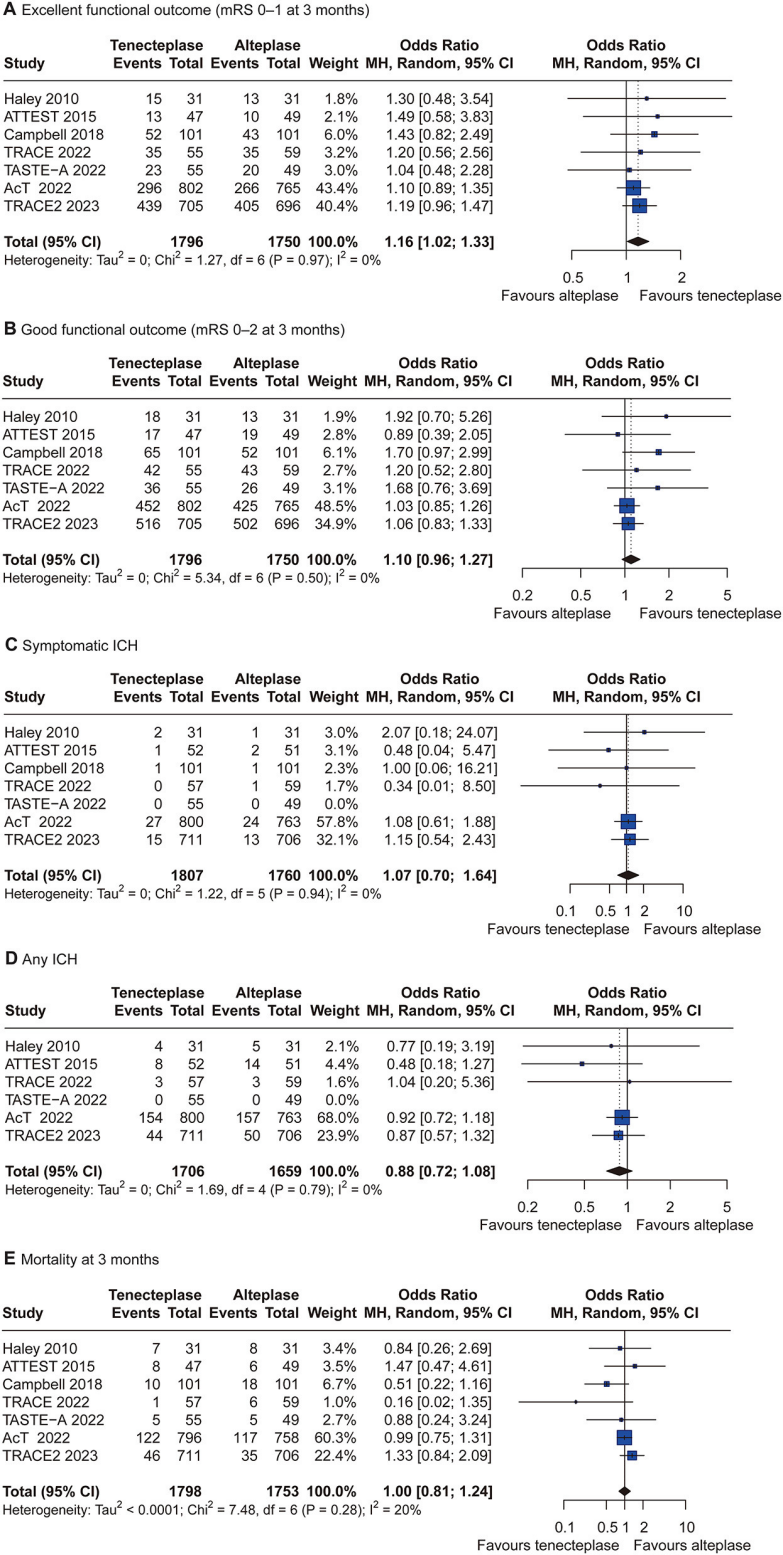
Forest plots of tenecteplase 0.25 mg/kg vs. alteplase 0.9 mg/kg for all outcomes. CI = confidence interval; ICH = intracranial hemorrhage. **(A)** excellent functional outcome; **(B)** good functional outcome; **(C)** symptomatic intracranial hemorrhage; **(D)** any intracranial hemorrhage; and **(E)** mortality at 3 months.

Given that a significant number of patients treated with tenecteplase 0.25 mg/kg and alteplase 0.9 mg/kg were included in this NMA, we performed a random-effects pairwise meta-analysis of tenecteplase 0.25 mg/kg vs. alteplase 0.9 mg/kg for the treatment of AIS within 4.5 h of symptom onset to obtain evidence of a direct comparison. Seven RCTs included 3,598 patients, of whom 1,818 were treated with tenecteplase 0.25 mg/kg, and 1,780 were treated with alteplase 0.9 mg/kg. The pairwise meta-analysis results indicated that tenecteplase 0.25 mg/kg had significantly higher odds of excellent functional outcome (OR, 1.16; 95% CI, 1.02–1.33; P = 0.03) compared with alteplase 0.9 mg/kg. No significant differences were found in good functional outcome (OR, 1.10; 95% CI, 0.96–1.27; *P* = 0.17) and safety outcomes (Figure 4). There was no significant heterogeneity across the studies for all outcomes.

## Discussion

In the present NMA, we evaluated the intervention effects of various doses of tenecteplase (0.1, 0.25, 0.32, and 0.4 mg/kg), alteplase 0.9 mg/kg, and placebo in AIS patients within 4.5 h of symptom onset. We discovered that the doses of 0.1, 0.25, and 0.32 mg/kg tenecteplase and 0.9 mg/kg alteplase were more likely to improve clinical outcomes than placebo. However, only the effect estimates of tenecteplase 0.25 mg/kg and alteplase 0.9 mg/kg revealed a statistically significant difference in excellent and good functional outcomes at 3 months. Furthermore, compared to alteplase 0.9 mg/kg, tenecteplase 0.25 mg/kg exhibited significantly higher odds of excellent functional outcome at 3 months. In terms of safety, the pooled results of our NMA indicated that various doses of tenecteplase (0.1, 0.25, 0.32, and 0.4 mg/kg) and alteplase (0.9 mg/kg) substantially increased the sICH risk compared with placebo. Moreover, compared to placebo, tenecteplase 0.32 mg/kg, tenecteplase 0.4 mg/kg, and alteplase 0.9 mg/kg not only significantly increased the risk of sICH but also significantly increased the risk of any ICH. However, mortality at 3 months did not differ significantly between the treatment regimens.

The SUCRA results of this NMA demonstrated that tenecteplase 0.25 mg/kg had the highest ranking among the stroke thrombolysis regimens regarding efficacy outcomes. In terms of safety, the SUCRA result of sICH revealed that placebo had the lowest risk of sICH, followed by alteplase 0.9 mg/kg and tenecteplase 0.25 mg/kg. SUCRA results also demonstrated that tenecteplase 0.25 mg/kg had the lowest risk of any ICH except placebo. On the contrary, tenecteplase 0.4 mg/kg was ranked lowest in efficacy outcomes and highest in the risk of ICH (including sICH and any ICH) and mortality at 3 months.

A phase III study, NOR-TEST-1, discovered that tenecteplase 0.4 mg/kg was safe but no better than alteplase 0.9 mg/kg (29). However, most patients in NOR-TEST-1 had mild strokes (median NIHSS = 4), so the results might not accurately reflect the actual effect of thrombolytic therapy on moderate or severe stroke patients. Moreover, the EXTEND-IA TNK Part II study did not demonstrate that 0.4 mg/kg improved cerebral reperfusion more than 0.25 mg/kg tenecteplase (30). In phase III, NOR TEST-2 trial (19) (median NIHSS = 11.5) and Phase IIB/III trial (24) (median NIHSS = 9), tenecteplase 0.4 mg/kg was early terminated because it resulted in a higher risk of sICH and had worse clinical outcomes than tenecteplase 0.25 mg/kg or alteplase 0.9 mg/kg. In our NMA, we discovered that fewer patients treated with tenecteplase 0.4 mg/kg had favorable clinical outcomes, and the prevalence of ICH was higher than those who received placebo. Two recent NMAs showed that, compared to alteplase 0.9 mg/kg, tenecteplase 0.4 mg/kg had a significantly higher risk of any parenchymal hematoma and a trend toward an increased risk of sICH, with no statistically significant difference in efficacy outcomes (31, 32). However, in our NMA, we found that tenecteplase 0.4 mg/kg had significantly worse clinical outcomes and statistically significantly increased the risk of ICH compared to tenecteplase 0.25 mg/kg and alteplase 0.9 mg/kg. Furthermore, based on SUCRA results, tenecteplase 0.4 mg/kg ranked as the worst treatment dose for all efficacy and safety outcomes in this NMA. We presented new evidence indicating that tenecteplase 0.4 mg/kg was not superior to alteplase 0.9 mg/kg or tenecteplase 0.25 mg/kg in improving clinical functional outcomes and posed a higher risk of ICH.

Although evidence from accumulated clinical trial data demonstrated that tenecteplase 0.25 mg/kg was as safe and effective as alteplase 0.9 mg/kg for AIS and may even provide better outcomes, the results remain inconclusive (33–36). A meta-analysis based on four RCTs involving 1,390 participants revealed that tenecteplase 0.25 mg/kg subgroup was associated with considerably greater early neurological improvement (*P* < 0.001) and a tendency toward a decreased risk of any ICH (*P* = 0.076) than alteplase 0.9 mg/kg (35). Another meta-analysis of six RCTs indicated no significant difference in early neurological improvement between tenecteplase 0.25 mg/kg and alteplase 0.9 mg/kg subgroups (*P* = 0.38) (36). However, these meta-analyses included the patients beyond and within 4.5 h of symptom onset. A mixed analysis of data from both time windows beyond and within 4.5 h may not accurately convey the strength of the evidence for the core indicator of thrombolysis within 4.5 h because benefit declines sharply with longer onsets to treatment time.

In the present NMA, compared with placebo, tenecteplase 0.25 mg/kg and alteplase 0.9 mg/kg significantly increased the rates of excellent and good functional outcomes, and these differences were statistically significant. Although there was a significantly increased risk of sICH, tenecteplase 0.25 mg/kg and alteplase 0.9 mg/kg did not differ significantly in mortality at 3 months compared to placebo. The NMA results demonstrated that tenecteplase 0.25 mg/kg and alteplase 0.9 mg/kg were safe and more effectively improved clinical outcomes for AIS within 4.5 h of symptom onset.

A recent pairwise and NMA showed that tenecteplase 0.25 mg/kg was associated with significant improvement in early neurological improvement and excellent functional outcome at 3 months compared with alteplase 0.9 mg/kg (32). Similar to this finding, the pooled results of our study, which included the latest RCT (TRACE-2), also indicated that tenecteplase 0.25 mg/kg was superior to alteplase 0.9 mg/kg in excellent functional outcome (*P* = 0.03). In addition, no significant difference was observed in any ICH between tenecteplase 0.25 mg/kg and alteplase 0.9 mg/kg in the two recent meta-analyses (31, 32). However, our NMA found that there was no significant difference between tenecteplase 0.25 mg/kg and placebo, whereas there was a statistically significant difference between alteplase 0.9 mg/kg and placebo in any ICH. The results from using placebo as the reference treatment suggested that tenecteplase 0.25 mg/kg had a lower risk of any ICH than alteplase 0.9 mg/kg.

Our NMA pooled results and the SUCRA ranking for efficacy and safety outcomes tended to support that tenecteplase 0.25 mg/kg has a better benefit-risk balance for thrombolytic therapy in AIS within 4.5 h of symptom onset. Moreover, tenecteplase 0.25 mg/kg demonstrated superiority over alteplase 0.9 mg/kg in terms of excellent functional outcome and had a lower risk of any ICH, suggesting that tenecteplase 0.25 mg/kg may offer greater benefit than alteplase 0.9 mg/kg and has the potential to replace alteplase 0.9 mg/kg in AIS treatment. Currently, tenecteplase 0.25 mg/kg is being evaluated in phase III clinical trials, including the TASTE trial (Registration number: ACTRN126131000243718) (37) and the ATEST 2 trial (ClinicalTrials.gov Identifier: NCT02814409) (38), which may provide further evidence of the effectiveness of tenecteplase 0.25 mg/kg in the treatment of AIS within 4.5 h of symptom onset.

### Limitations of this study

Several limitations must be considered in this NMA. First, we placed great emphasis on RCT. Thus, we may ignore potentially useful information from nonrandomized studies. Although this is a weakness, it is also a strength because it focuses only on the kinds of articles with the highest evidence hierarchy. Second, the sICH definition used in different studies may differ from the one used herein, but when applied to our data, it may affect the frequency of bleeding. In addition, because there were zero sICH events in some treatment arms of three studies, leading to large ORs and wide 95% CrI, these results should be interpreted cautiously. Third, there was low heterogeneity between studies, and a potential source of heterogeneity may be the slightly different inclusion and exclusion criteria between studies. Fourth, SUCRA results should be interpreted cautiously since high rankings may only offer suggestive rather than conclusive evidence for treatment choices. Finally, it is difficult to assess publication bias in an NMA because of the limited number of papers in each pairwise comparison.

## Conclusions

The NMA indicated that tenecteplase 0.25 mg/kg and alteplase 0.9 mg/kg are safe and significantly improve clinical outcomes in patients with AIS within 4.5 h of symptom onset. Furthermore, tenecteplase 0.25 mg/kg appears to provide more benefit than alteplase 0.9 mg/kg and has the potential to replace alteplase 0.9 mg/kg in AIS treatment. However, given several limitations of this study, further research is required to confirm the findings.

## Data availability statement

The original contributions presented in the study are included in the article/Supplementary material, further inquiries can be directed to the corresponding authors.

## Author contributions

HL, QH, and SL reviewed abstracts and titles of potentially relevant studies and screened full-text articles. XW and BQ extracted all the data independently. HL and XQ verified the extracted data. XQ and SC independently evaluated the risk of bias in the included studies. HL and XW performed all statistical analyses, drafted the manuscript, and made critical revisions. XQ and JZ contributed to the study's design and revised the manuscript. ZL reviewed the manuscript critically. All authors contributed to the article and approved the submitted version.
