# Supplementary material for: Different doses of tenecteplase vs. alteplase for acute ischemic stroke within 4.5 hours of symptom onset: a network meta-analysis of randomized controlled trials
Source: Front Neurol. 2023 Jun 2;14:1176540. doi: 10.3389/fneur.2023.1176540 (PMC10274135; doi:10.3389/fneur.2023.1176540)
Supplement: Supplementary file 1 [file Data_Sheet_1.pdf]

Supplementary Material

Different Doses of Tenecteplase versus Alteplase for Acute Ischemic Stroke within 4.5 Hours of Symptom Onset: A Network Meta-Analysis of Randomized Controlled Trials

Huo Liang[1,†], Xue Wang[1,†], Xuemei Quan[2], Shijian Chen[1], Bin Qin[1], Shuolin Liang[1], Qiuhui Huang[1] , Jian Zhang[1,*], Zhijian Liang[1,*]

1 Department of Neurology, The First Affiliated Hospital of Guangxi Medical University, Nanning, 530021, China

2 Department of Neurology, The People's Hospital of Guangxi Zhuang Autonomous Region, Nanning, 530021, China

[†] These authors contributed equally to this work and share first authorship

[*]Zhijian Liang and Jian Zhang are co–corresponding authors.

Correspondence Author: Zhijian Liang, Department of Neurology, The First Affiliated Hospital of Guangxi Medical University, Nanning, 530021, China & Guangxi Key Laboratory of Precision Medicine in Cardio-cerebrovascular Diseases Control and Prevention & Guangxi Clinical Research Center for Cardio-cerebrovascular Diseases, Nanning, China, **Email** liangzhijian@gxmu.edu.cn. Jian Zhang, Department of Neurology, The First Affiliated Hospital of Guangxi Medical University, Nanning, 530021, China, **Email** Janzu@qq.com.

Supplementary material included:

Supplementary Tables (Table 1- Table 3)

Supplementary Figures (Figure 1-Figure 13)

Supplementary Table 1. Search strategy

PubMed (1041 results):
((("Tenecteplase"[MeSH Terms] OR "Tenecteplase"[Title/Abstract] OR "TNK-tPA"[Title/Abstract] OR "TNK"[Title/Abstract] OR "Metalyse"[Title/Abstract] OR "TNKase"[Title/Abstract] OR "recombinant human tnk tissue type plasminogen activator"[Title/Abstract] OR "rhTNK-tPA"[Title/Abstract] OR ("tissue plasminogen activator"[MeSH Terms] OR "tissue plasminogen activator"[Title/Abstract] OR "tissue type plasminogen activator"[Title/Abstract] OR "tissue type plasminogen activator"[Title/Abstract] OR "t plasminogen activator"[Title/Abstract] OR "t plasminogen activator"[Title/Abstract] OR "Alteplase"[Title/Abstract] OR "plasminogen activator tissue type"[Title/Abstract] OR "plasminogen activator tissue type"[Title/Abstract] OR "rt-PA"[Title/Abstract])) AND ("Stroke"[MeSH Terms] OR "Stroke"[Title/Abstract] OR "cerebrovascular accident"[Title/Abstract] OR "brain vascular accident"[Title/Abstract] OR "acute stroke"[Title/Abstract]) AND ("randomized controlled trial"[Publication Type] OR "controlled clinical trial"[Publication Type] OR "random*"[Title/Abstract] OR "RCT"[Title/Abstract])) NOT ("Meta-Analysis"[Publication Type] OR "Review"[Publication Type] OR "systematic review"[Publication Type])) AND (humans[Filter])
Embase (1382 results):
No. Query Results<br>#12 #3 AND #10 AND ([randomized controlled trial]/lim OR 'controlled clinical trial'/de) AND [humans]/lim<br>#11 #3 AND #10<br>#10 #6 OR #9<br>#9 #7 OR #8<br>#8 'tissue plasminogen activator':ab,ti OR 'tissue-type plasminogen activator':ab,ti OR 'tissue type plasminogen activator':ab,ti OR 't-plasminogen activator':ab,ti OR 't plasminogen activator':ab,ti OR alteplase:ab,ti OR 'plasminogen activator, tissue-type':ab,ti OR 'plasminogen activator, tissue type':ab,ti OR 'rt pa':ab,ti<br>#7 'tissue plasminogen activator'/exp<br>#6 #4 OR #5<br>#5 tenecteplase:ab,ti OR 'tnk tpa':ab,ti OR tnk:ab,ti OR metalyse:ab,ti OR tnkase:ab,ti OR 'recombinant human tnk tissue-type plasminogen activator':ab,ti OR 'rhtnk tpa':ab,ti<br>#4 'tenecteplase'/exp<br>#3 #1 OR #2<br>#2 stroke:ab,ti OR 'cerebrovascular accident':ab,ti OR 'brain vascular accident':ab,ti OR 'acute stroke':ab,ti<br>#1 'cerebrovascular accident'/exp
Web of Science (3203 results):
TS=(Stroke OR Strokes OR Cerebrovascular Accident OR Cerebrovascular Accidents) AND (TS=(Tissue Plasminogen Activator OR Plasminogen Activator, Tissue OR Tissue Activator D-44 OR Tissue Activator D 44 OR Tisokinase OR Tissue-Type Plasminogen Activator OR Tissue Type Plasminogen Activator OR TTPA OR T-Plasminogen Activator OR T Plasminogen Activator OR Alteplase OR Plasminogen Activator, Tissue-Type OR Plasminogen Activator, Tissue Type OR Activase OR Actilyse OR Lysatec rt-PA OR Lysatec rt PA OR Lysatec rtPA) OR TS=(Tenecteplase OR TNK-tPA OR TNK OR Metalyse OR TNKase OR Recombinant human TNK tissue type plasminogen activator* OR rhTNK-tPA)) AND TS=(Randomized Controlled Trial OR RCT OR Random*)
Cochrane library (710 results):
#1 MeSH descriptor: [Tenecteplase] explode all trees<br>#2 (Tenecteplase OR TNK-tPA OR TNK OR Metalyse OR TNKase OR recombinant human

TNK tissue-type plasminogen activator OR rhTNK-tPA):ti,ab,kw (Word variations have been searched)
#3 MeSH descriptor: [Tissue Plasminogen Activator] explode all trees
#4 (Tissue Plasminogen Activator OR Tissue-Type Plasminogen Activator OR Tissue Type Plasminogen Activator OR T-Plasminogen Activator OR T Plasminogen Activator OR Alteplase OR Plasminogen Activator, Tissue-Type OR Plasminogen Activator, Tissue Type OR rt-PA):ti,ab,kw (Word variations have been searched)
#5 #1 OR #2 OR #3 OR #4
#6 MeSH descriptor: [Stroke] explode all trees
#7 (stroke OR Cerebrovascular Accident OR Brain Vascular Accident OR Acute Stroke):ti,ab,kw (Word variations have been searched)
#8 #6 OR #7
#9 (controlled clinical trial):pt (Word variations have been searched)
#10 (randomized controlled trial):pt (Word variations have been searched)
#11 #9 OR #10
#12 #5 AND #8 AND #11

Supplementary Table 2. The heterogeneity test of NMA

Outcomes	Global I-squared (%)	
	I^2.pair	I^2.cons
Excellent functional outcome	0	0
Good functional outcome	0	0
Symptomatic intracranial hemorrhage	0	0
Any intracranial hemorrhage	16.35	0
Mortality at 3 months	28.84	15.04

NMA = network meta-analysis; I^2.pair = I^2 of pair-wise meta-analysis; I^2.cons = I^2 of network meta-analysis; NA = not available.

Supplementary Table 3. The ranking of treatments for each outcome

Treatments (mg/kg)	versus Placebo<br>OR (95% CrI)	SUCRA values	Ranking
A. Excellent functional outcome			
TNK 0.25	**1.85 (1.44, 2.37)**	0.87	1
TNK 0.32	1.81 (0.93, 3.58)	0.76	2
ALT 0.9	**1.60 (1.29, 1.97)**	0.61	3
TNK 0.1	1.40 (0.81, 2.44)	0.50	4
Placebo	-	0.19	5
TNK 0.4	0.80 (0.46, 1.37)	0.06	6
B. Good functional outcome			
TNK 0.25	**1.54 (1.19, 1.98)**	0.88	1
ALT 0.9	**1.40 (1.14, 1.74)**	0.68	2
TNK 0.32	1.27 (0.63, 2.66)	0.58	3
TNK 0.1	1.24 (0.69, 2.22)	0.55	4
Placebo	-	0.29	5
TNK 0.4	0.62 (0.35, 1.07)	0.02	6
C. Symptomatic ICH			
TNK 0.4	**110.21 (13.97, 1354.02)**	0.96	1
TNK 0.32	**26.11 (1.51, 479.94)**	0.61	2
TNK 0.1	**21.79 (2.04, 245.99)**	0.57	3
TNK 0.25	**14.65 (3.25, 101.67)**	0.44	4
ALT 0.9	**14.14 (4.00, 86.26)**	0.42	5
Placebo	-	< 0.01	6
D. Any ICH			
TNK 0.4	**8.37 (2.72, 39.54)**	0.93	1
TNK 0.32	**4.66 (1.08, 29.62)**	0.71	2
TNK 0.1	3.37 (0.98, 16.18)	0.57	3
ALT 0.9	**2.54 (1.45, 8.08)**	0.46	4
TNK 0.25	2.13 (0.98, 7.85)	0.31	5
Placebo	-	0.02	6
E. Mortality at 3 months			
TNK 0.4	1.80 (0.47, 7.38)	0.84	1
TNK 0.32	1.02 (0.19, 6.41)	0.50	2
ALT 0.9	0.98 (0.50, 2.67)	0.49	3
Placebo	-	0.49	4
TNK 0.25	0.92 (0.37, 2.76)	0.41	5
TNK 0.1	0.73 (0.19, 3.30)	0.32	6

For efficacy outcomes (A) and (B), the first ranking is the best efficacy. For safety outcomes (C, D, and E), the opposite was true. For example, TNK 0.25 mg/kg ranked best for improving efficacy outcomes, whereas TNK 0.4 mg/kg ranked poorest. SUCRA = surface under the cumulative ranking curve; TNK = tenecteplase; ALT = alteplase; ICH = intracranial hemorrhage.

Supplementary Figure 1–5. Trace plots to evaluate model convergence

Supplementary Figure 1. Convergence: Excellent functional outcome

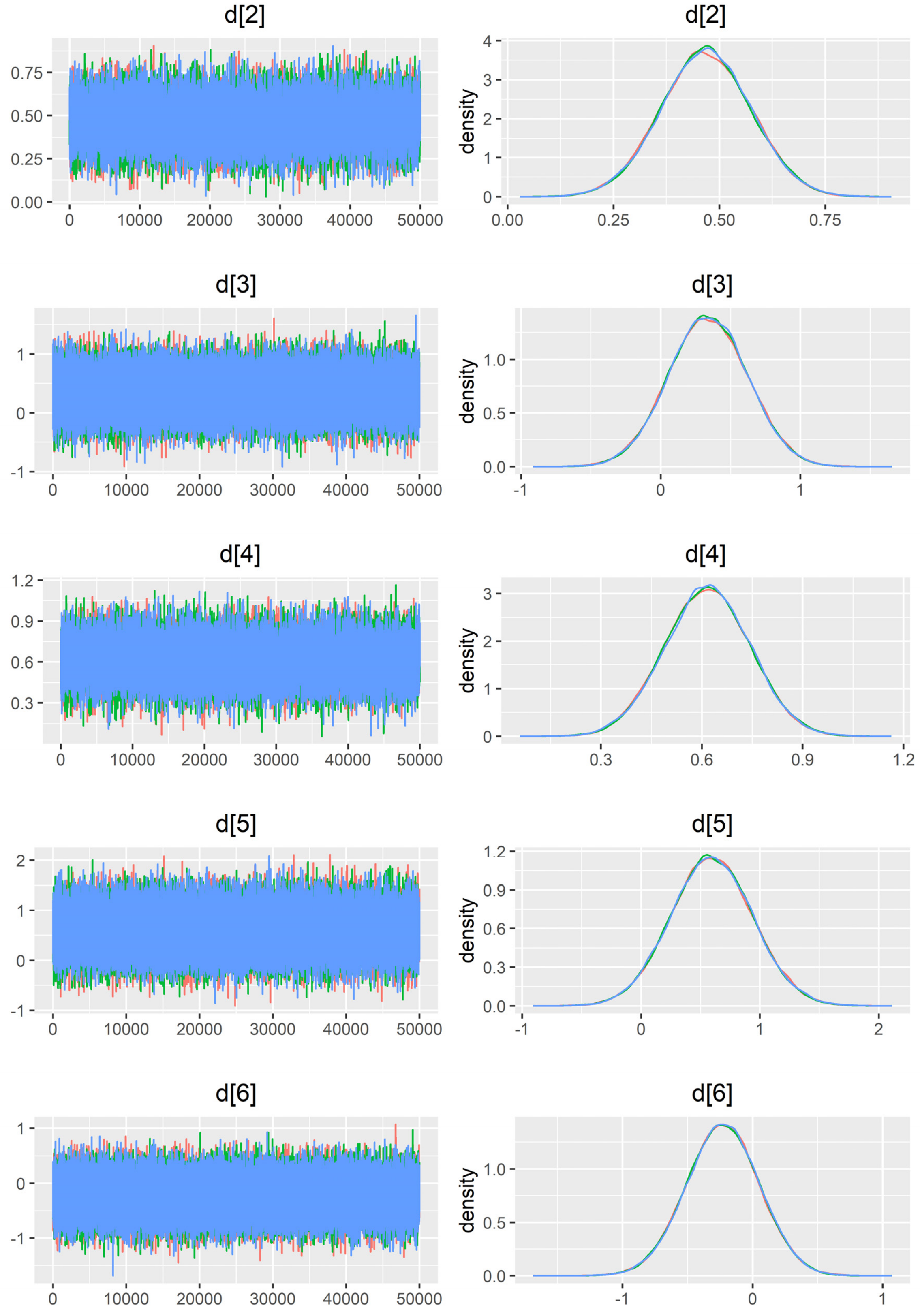

Supplementary Figure 2. Convergence: Good functional outcome

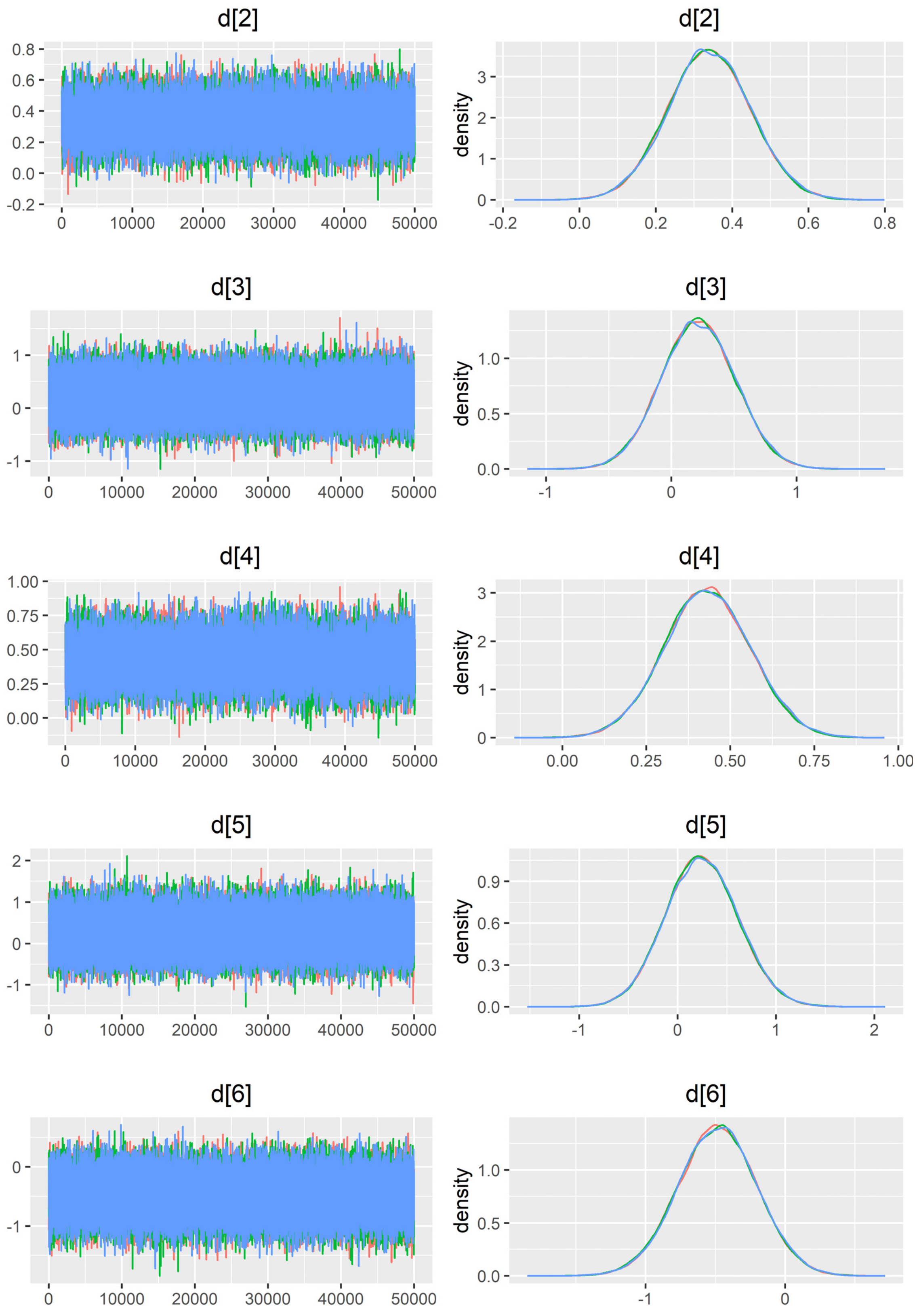

Supplementary Figure 3. Convergence: Symptomatic intracranial hemorrhage

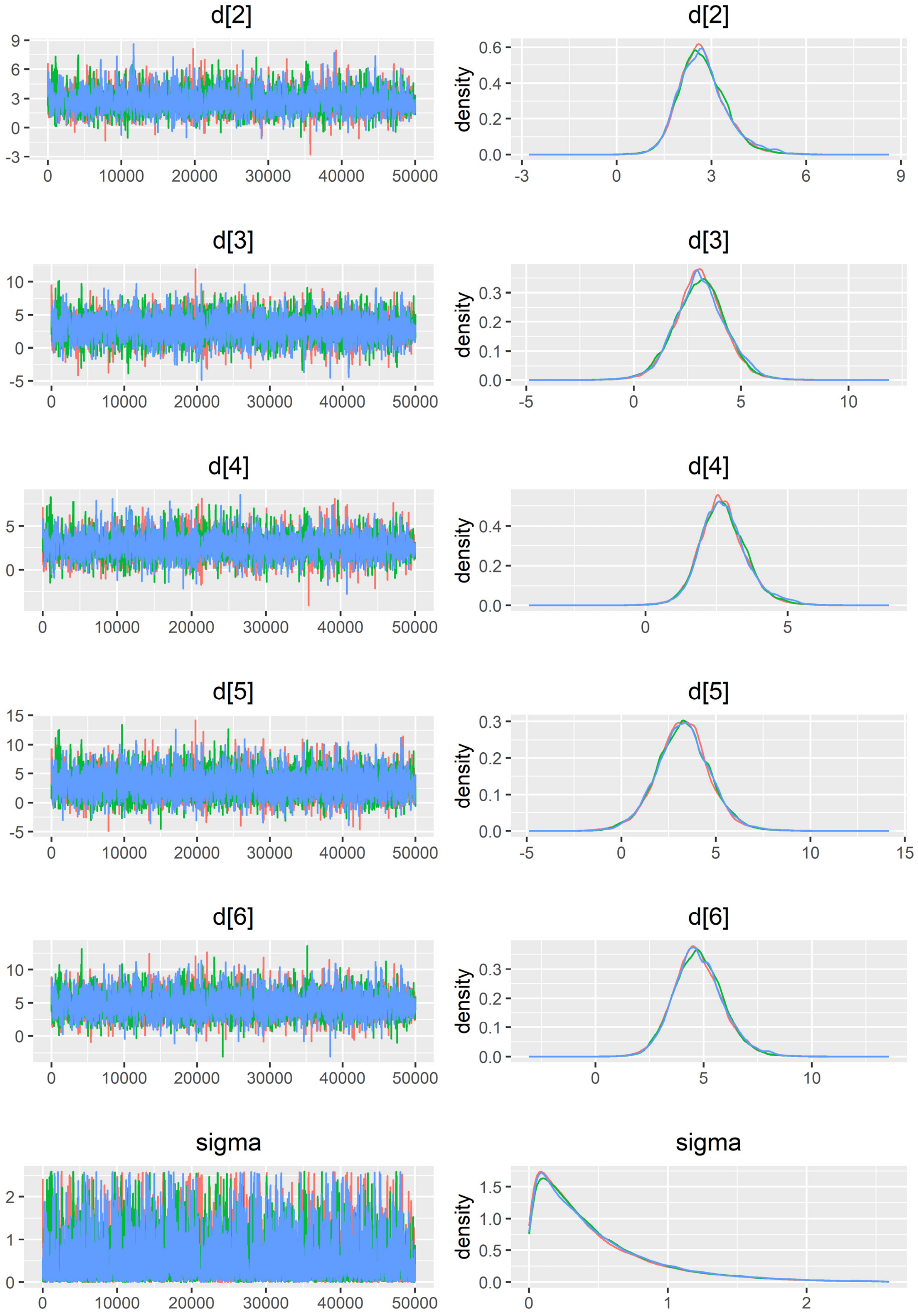

Supplementary Figure 4. Convergence: Any intracranial hemorrhage

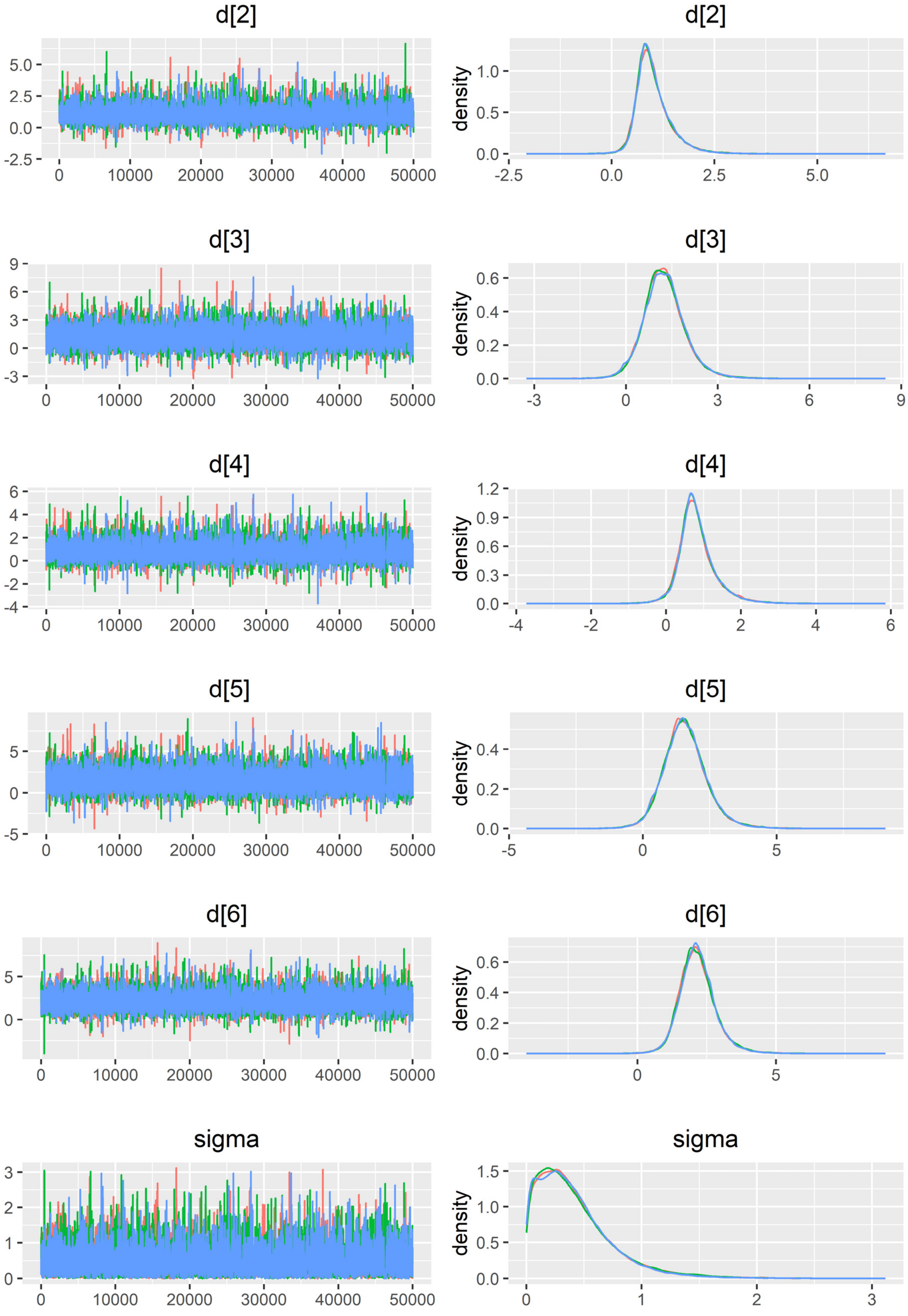

Supplementary Figure 5. Convergence: mortality at 3 months

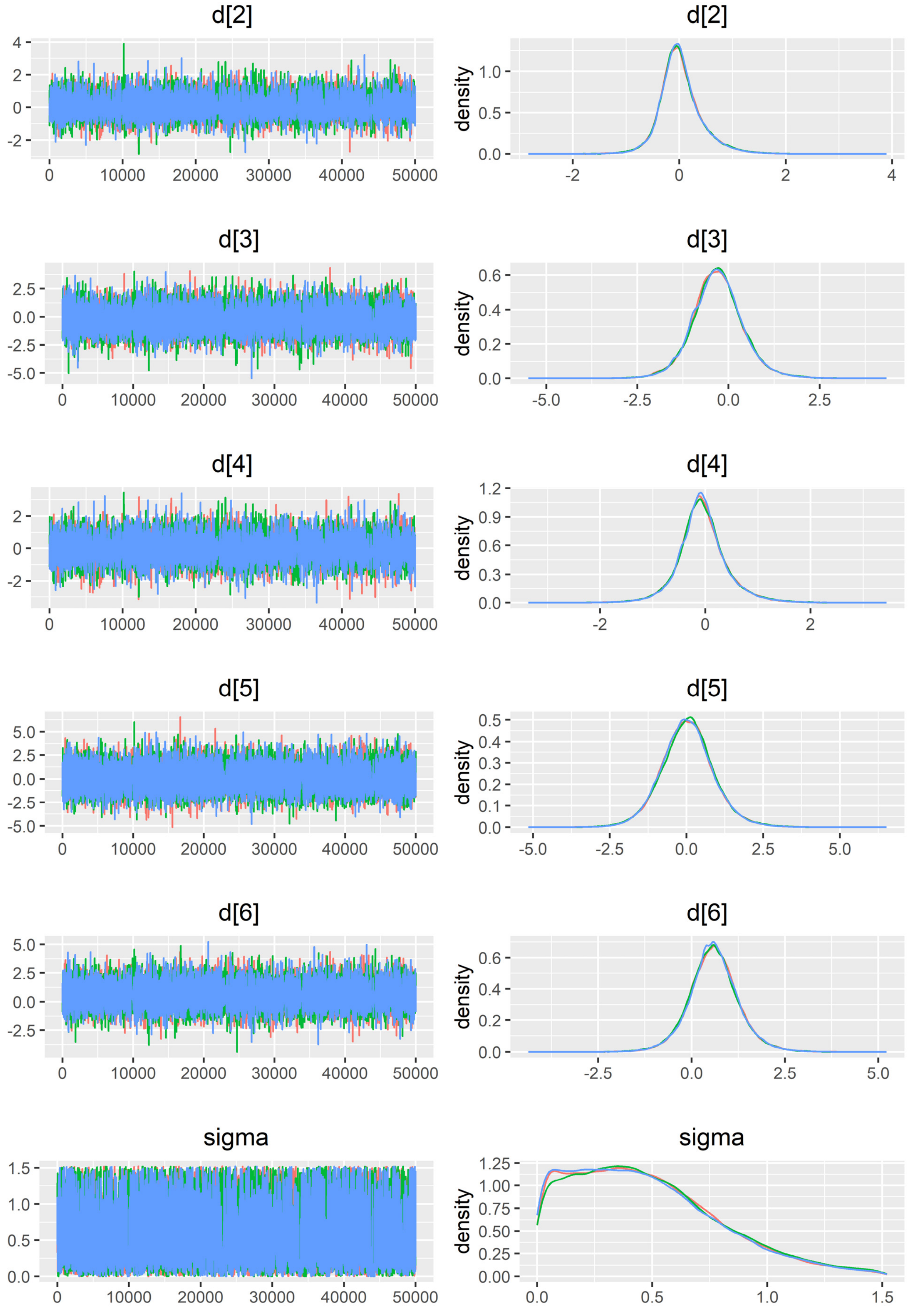

Supplementary Figure 6. PRISMA2020 flow diagram

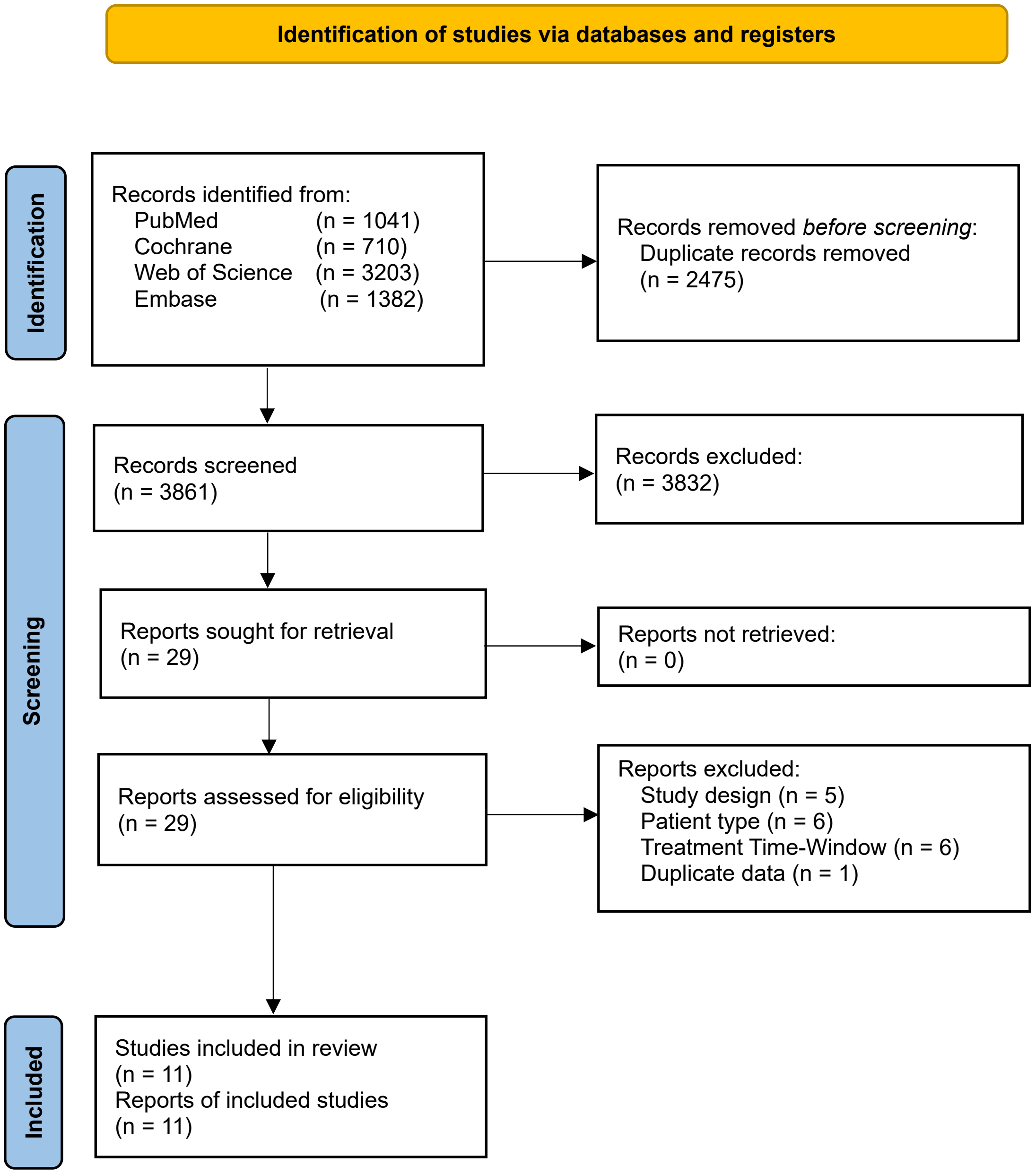

Supplementary Figure 7. Network diagrams for all outcomes

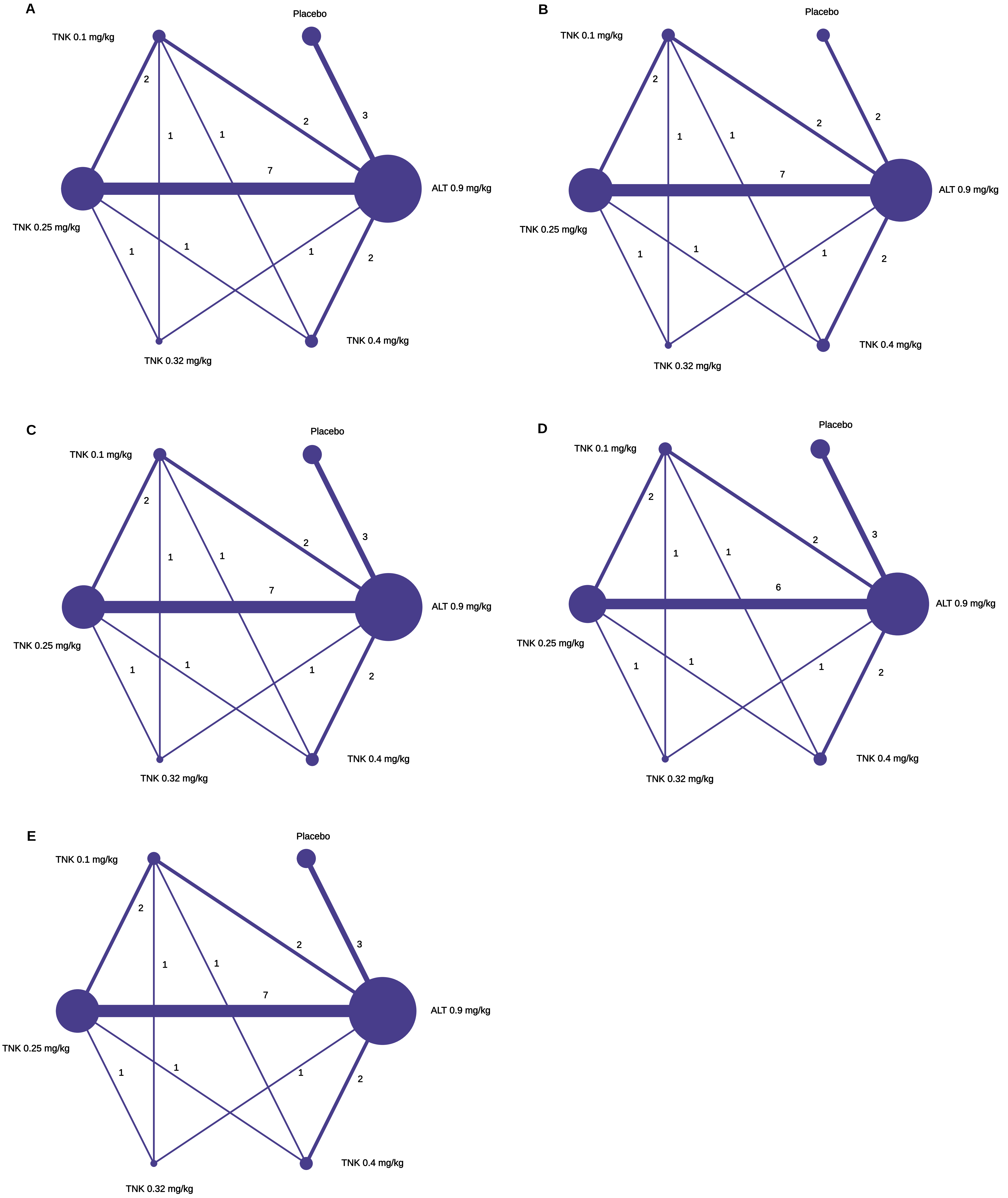

Legend: Each node represents a treatment, whereas the lines between nodes reflect direct comparisons between treatment pairs. The size of each node is proportional to the number of intervention studies. The line width is related to the number of studies contributing to each direct comparison, and the number of studies is also presented on the line. (A) excellent functional outcome; (B) good functional outcome; (C) symptomatic intracranial hemorrhage; (D) any intracranial hemorrhage; and (E) mortality at 3 months. TNK = tenecteplase; ALT = alteplase.

Supplementary Figure 8. Risk of bias assessment for each eligible study

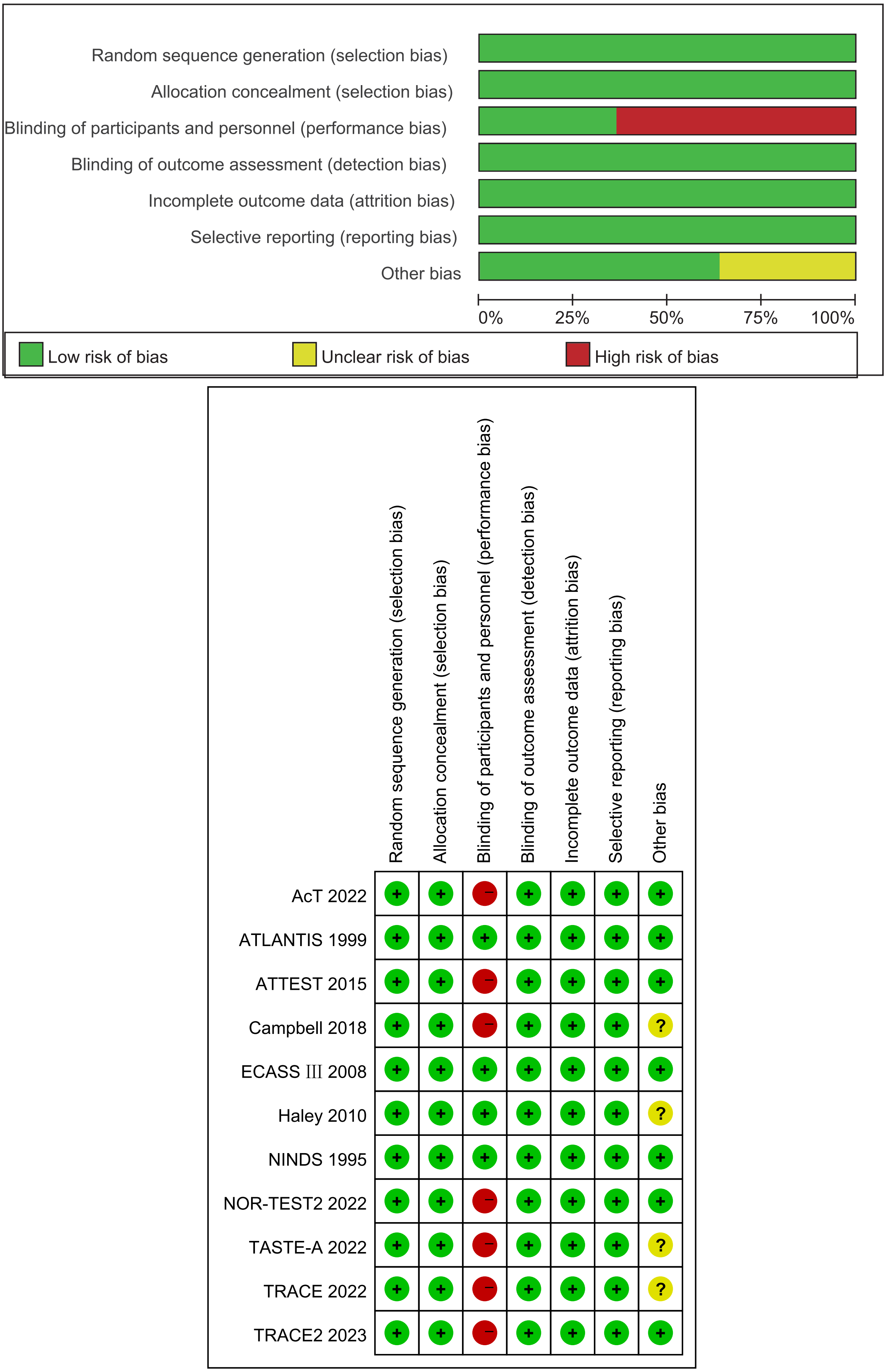

Supplementary Figure 9. Node-splitting analysis of inconsistency

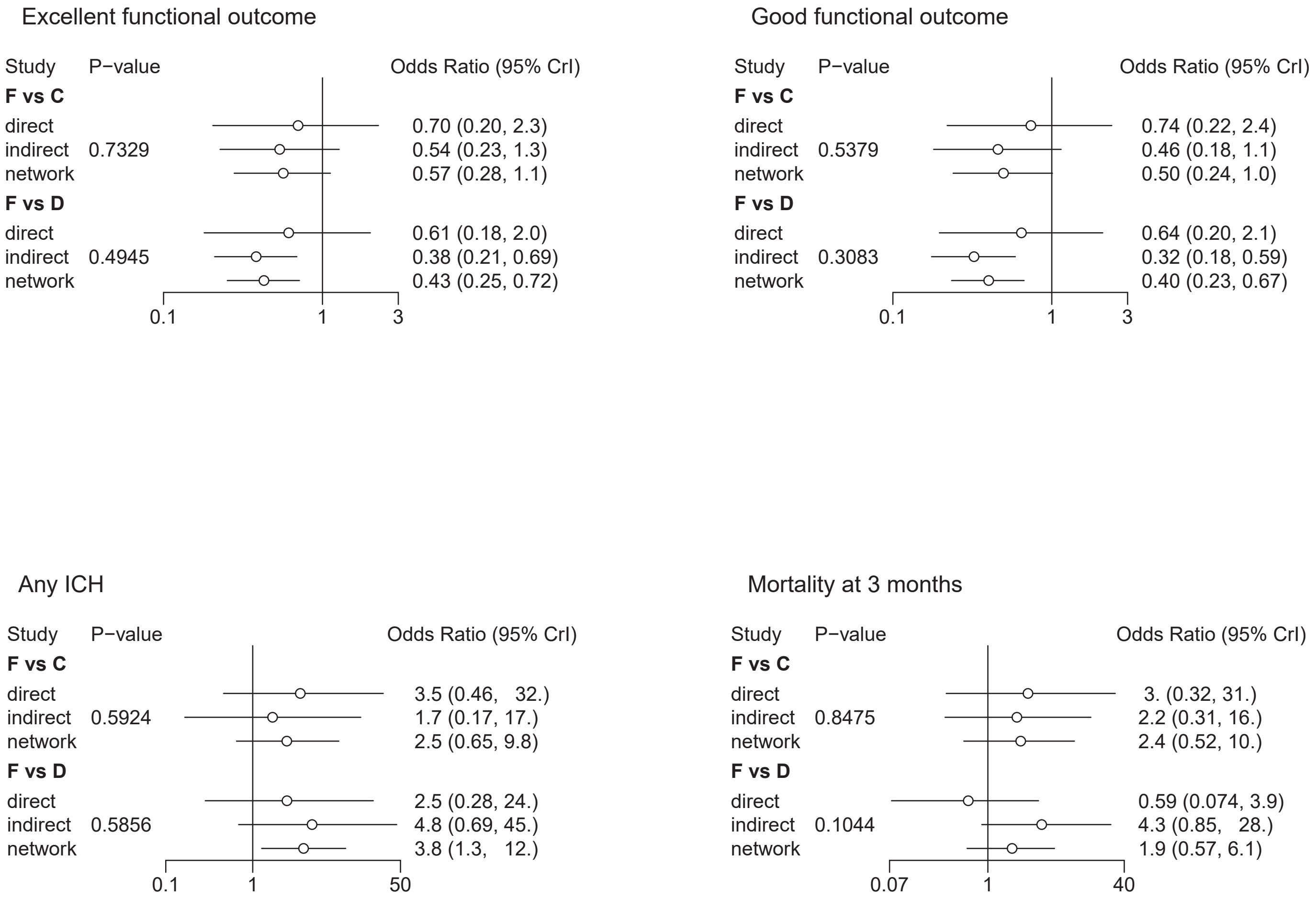

CrI = credible interval; C = tenecteplase 0.1 mg/kg; D = tenecteplase 0.25 mg/kg; F = tenecteplase 0.4 mg/kg.

Supplementary Figure 10. Leverage plots of inconsistency versus consistency models

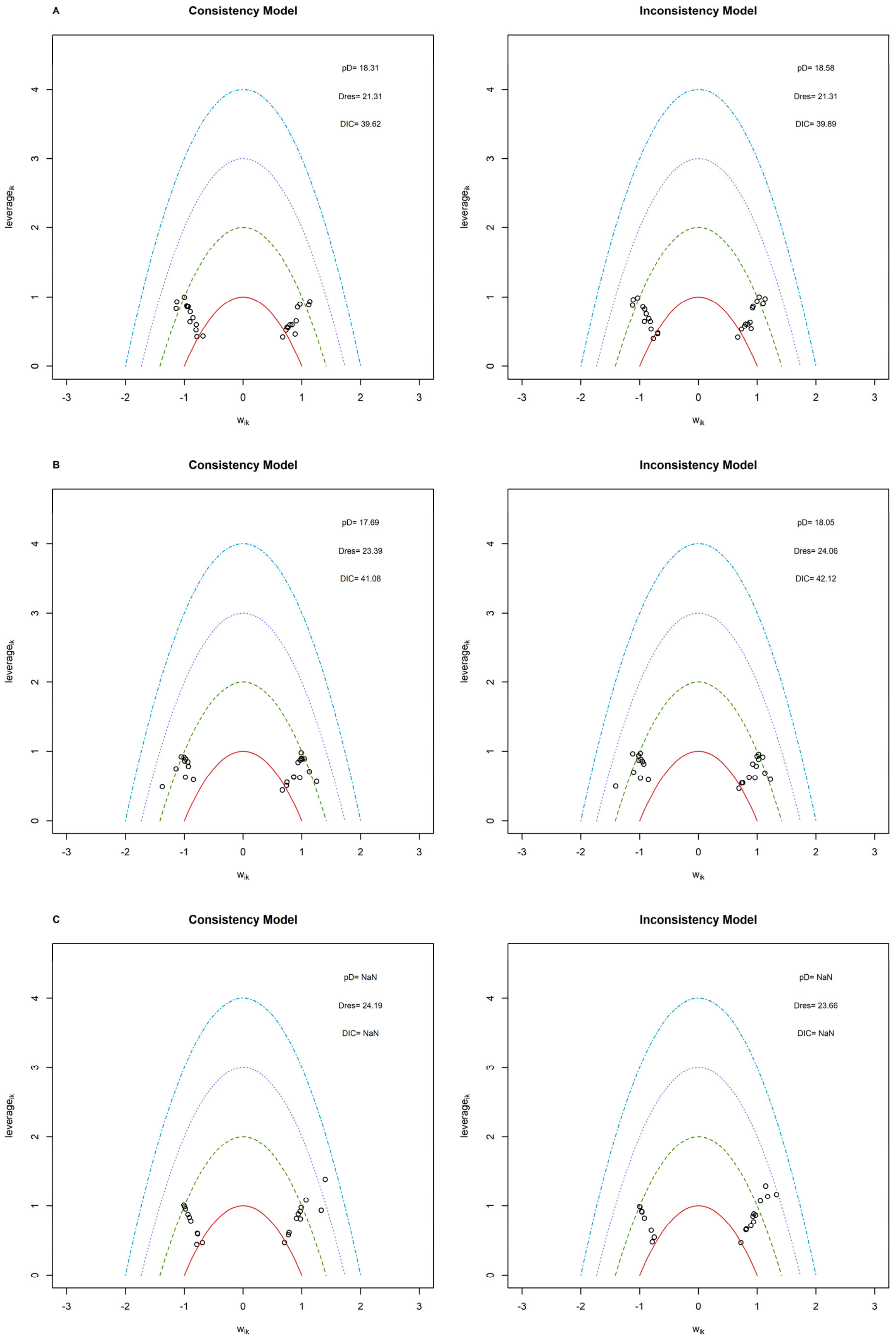

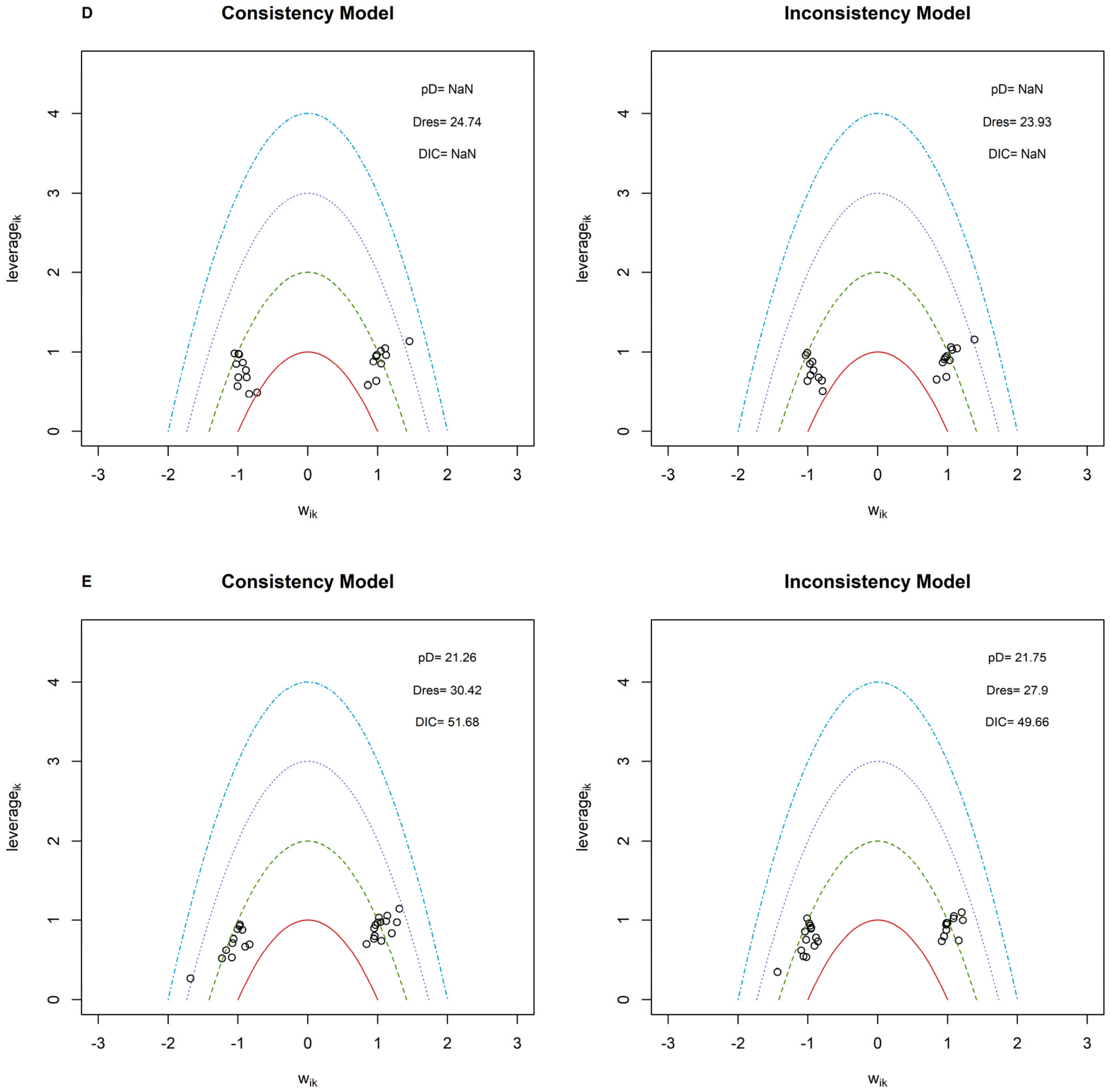

(A) excellent functional outcome; (B) good functional outcome; (C) symptomatic intracranial hemorrhage; (D) any intracranial hemorrhage; and (E) mortality at 3 months. DIC = deviance information criterion; Dres = posterior mean of the residual deviance; pD = effective number of parameters; NaN = not a number.

Supplementary Figure 11. Posterior mean deviance comparison plots

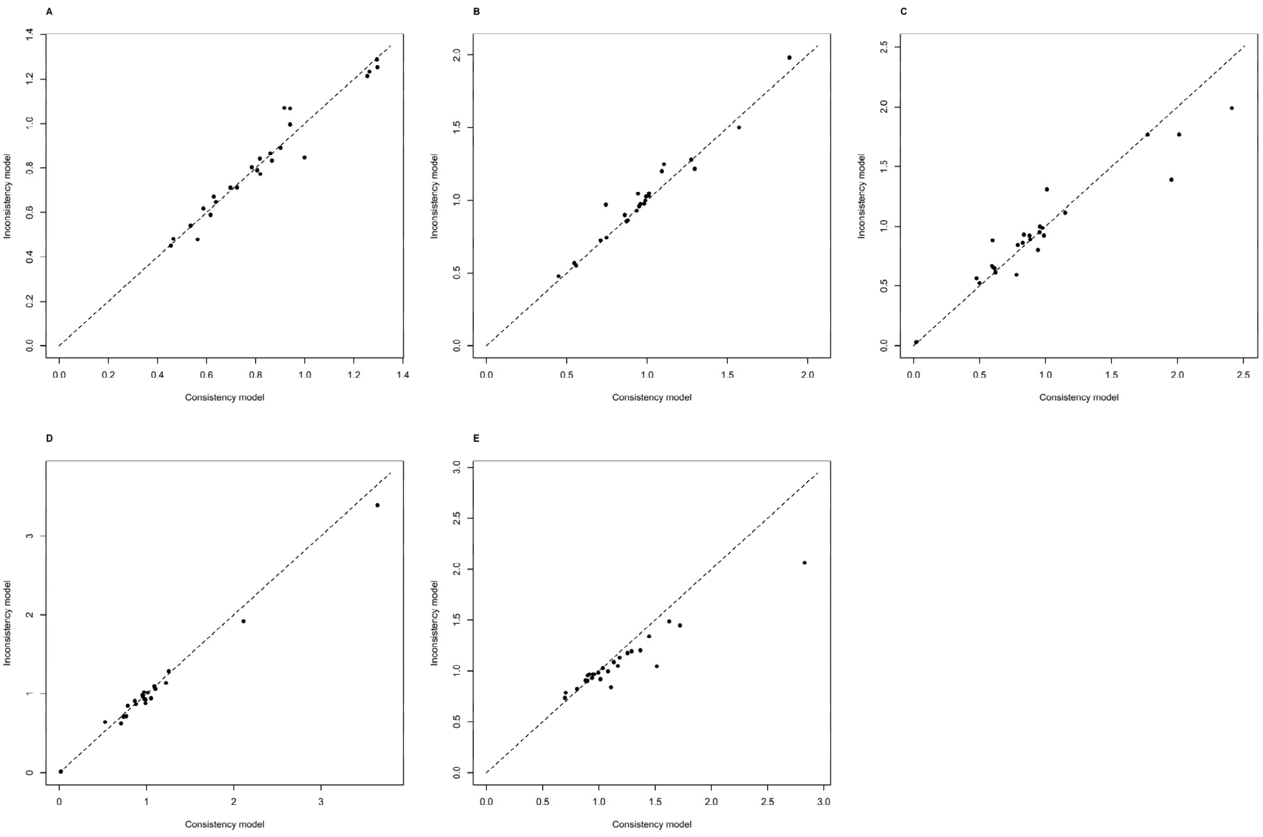

Legend: Each data point represents the contribution of a treatment arm to the posterior mean deviance for the consistency model and the inconsistency model. (A) excellent functional outcome; (B) good functional outcome; (C) symptomatic intracranial hemorrhage; (D) any intracranial hemorrhage; and (E) mortality at 3 months.

Supplementary Figure12. Leverage plots of fixed effects and random effects models

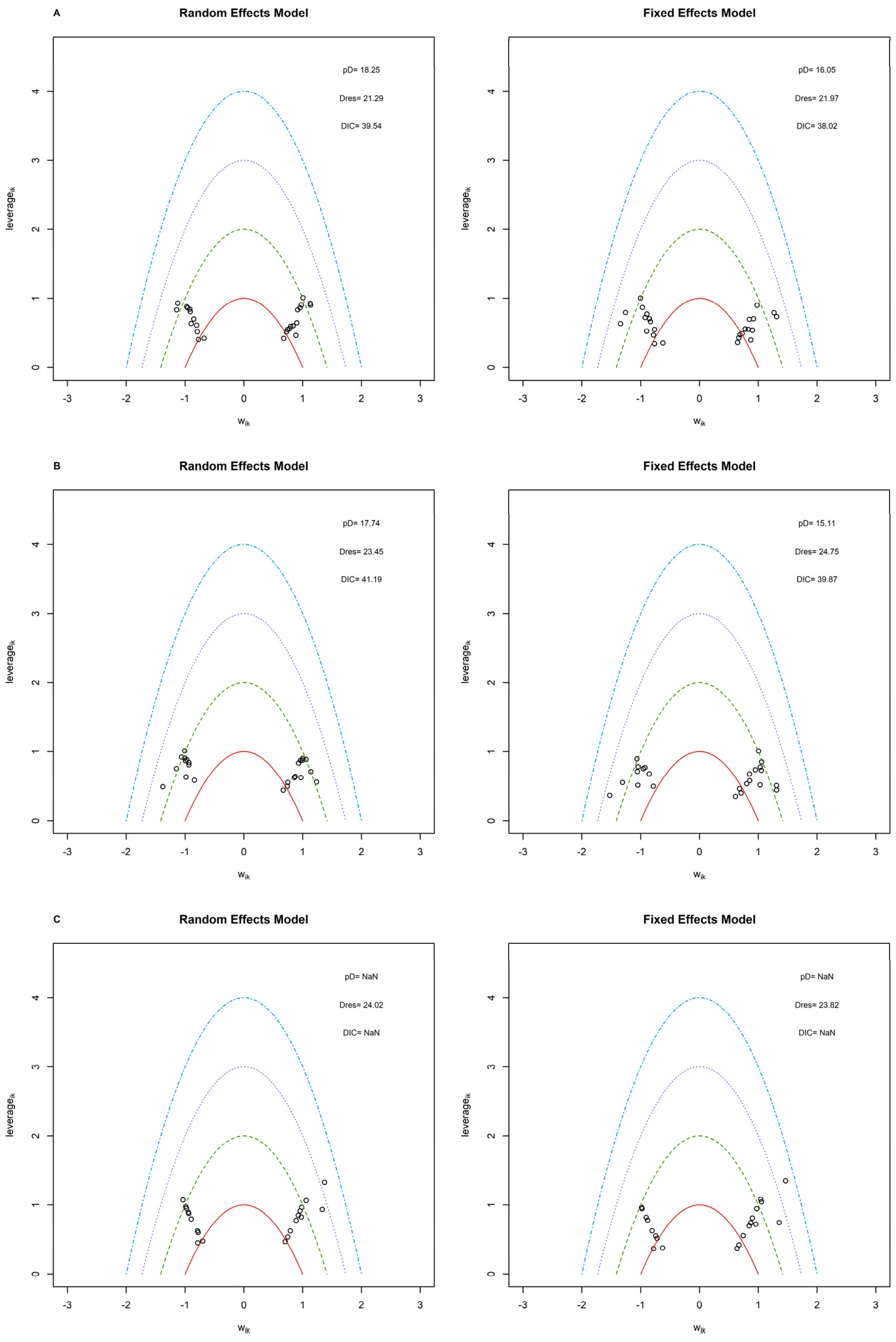

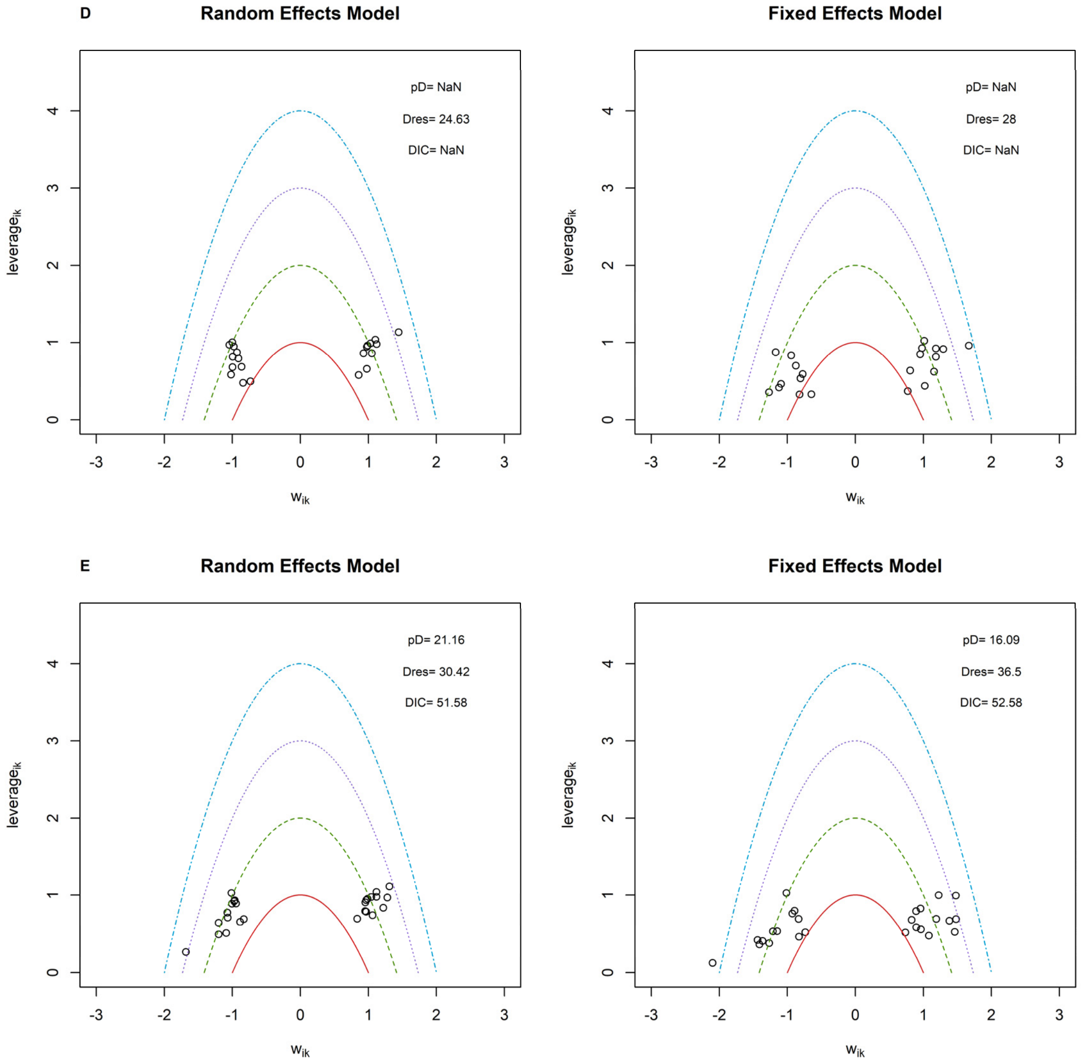

(A) excellent functional outcome; (B) good functional outcome; (C) symptomatic intracranial hemorrhage; (D) any intracranial hemorrhage; and (E) mortality at 3 months. DIC = deviance information criterion; Dres = posterior mean of the residual deviance; pD = effective number of parameters; NaN = not a number.

Supplementary Figure 13. League table heatmaps for safety outcomes

A

Treatment (columns) vs. Comparator (rows)

Comparator	TNK 0.4 mg/kg	TNK 0.32 mg/kg	TNK 0.1 mg/kg	TNK 0.25 mg/kg	ALT 0.9 mg/kg	Placebo
TNK 0.4 mg/kg		0.23 (0.01, 3.48)	0.19 (0.02, 1.53)	**0.13** (0.02, 0.72)	**0.13** (0.02, 0.66)	**0.01** (0.00, 0.07)
TNK 0.32 mg/kg	4.32 (0.29, 76.70)		0.84 (0.07, 9.35)	0.59 (0.05, 6.80)	0.57 (0.06, 6.65)	**0.04** (0.00, 0.66)
TNK 0.1 mg/kg	5.16 (0.65, 52.78)	1.19 (0.11, 14.09)		0.68 (0.12, 4.91)	0.66 (0.12, 4.88)	**0.05** (0.00, 0.49)
TNK 0.25 mg/kg	**7.41** (1.39, 48.90)	1.71 (0.15, 18.46)	1.46 (0.20, 8.59)		0.96 (0.46, 2.38)	**0.07** (0.01, 0.31)
ALT 0.9 mg/kg	**7.62** (1.52, 45.98)	1.76 (0.15, 18.01)	1.52 (0.20, 8.44)	1.04 (0.42, 2.19)		**0.07** (0.01, 0.25)
Placebo	**110.21** (13.97, 1354.02)	**26.11** (1.51, 479.94)	**21.79** (2.04, 245.99)	**14.65** (3.25, 101.67)	**14.14** (4.00, 86.26)	

B

Treatment (columns) vs. Comparator (rows)

Comparator	TNK 0.4 mg/kg	TNK 0.32 mg/kg	TNK 0.1 mg/kg	ALT 0.9 mg/kg	TNK 0.25 mg/kg	Placebo
TNK 0.4 mg/kg		0.56 (0.10, 3.00)	0.40 (0.10, 1.53)	**0.31** (0.11, 0.90)	**0.26** (0.08, 0.79)	**0.12** (0.03, 0.37)
TNK 0.32 mg/kg	1.80 (0.33, 9.92)		0.72 (0.17, 3.11)	0.56 (0.14, 2.35)	0.47 (0.11, 1.94)	**0.21** (0.03, 0.92)
TNK 0.1 mg/kg	2.49 (0.65, 9.86)	1.39 (0.32, 6.06)		0.78 (0.27, 2.44)	0.65 (0.21, 2.03)	0.30 (0.06, 1.02)
ALT 0.9 mg/kg	**3.20** (1.11, 8.71)	1.78 (0.43, 7.11)	1.28 (0.41, 3.72)		0.85 (0.44, 1.48)	**0.39** (0.12, 0.69)
TNK 0.25 mg/kg	**3.83** (1.26, 11.77)	2.13 (0.51, 8.86)	1.54 (0.49, 4.69)	1.18 (0.68, 2.29)		0.47 (0.13, 1.02)
Placebo	**8.37** (2.72, 39.54)	**4.66** (1.08, 29.62)	3.37 (0.98, 16.18)	**2.54** (1.45, 8.08)	2.13 (0.98, 7.85)	

C

Treatment (columns) vs. Comparator (rows)

Comparator	TNK 0.4 mg/kg	TNK 0.32 mg/kg	Placebo	ALT 0.9 mg/kg	TNK 0.25 mg/kg	TNK 0.1 mg/kg
TNK 0.4 mg/kg		0.57 (0.09, 3.79)	0.56 (0.14, 2.11)	0.56 (0.19, 1.83)	0.52 (0.16, 1.75)	0.41 (0.10, 1.85)
TNK 0.32 mg/kg	1.77 (0.26, 10.97)		0.98 (0.16, 5.32)	0.98 (0.22, 4.74)	0.91 (0.19, 4.36)	0.72 (0.14, 3.80)
Placebo	1.80 (0.47, 7.38)	1.02 (0.19, 6.41)		0.98 (0.50, 2.67)	0.92 (0.37, 2.76)	0.73 (0.19, 3.30)
ALT 0.9 mg/kg	1.79 (0.55, 5.13)	1.02 (0.21, 4.65)	1.02 (0.37, 2.02)		0.94 (0.50, 1.55)	0.74 (0.22, 2.26)
TNK 0.25 mg/kg	1.93 (0.57, 6.11)	1.10 (0.23, 5.27)	1.08 (0.36, 2.70)	1.06 (0.64, 2.00)		0.79 (0.24, 2.61)
TNK 0.1 mg/kg	2.46 (0.54, 10.35)	1.39 (0.26, 7.24)	1.36 (0.30, 5.37)	1.36 (0.44, 4.54)	1.27 (0.38, 4.18)	

Legend: League table heatmaps for safety outcomes. Data are ORs (95% CrI) of the treatment on the top, compared with the comparator on the left. OR < 1.0 shows an advantage for the treatment, whereas OR > 1.0 shows an advantage for the comparator. Statistically significant results ($P < 0.05$) are marked by the symbols (**). (A) symptomatic intracranial hemorrhage; (B) any intracranial hemorrhage; and (C) mortality at 3 months. OR = odds ratio; CrI = credible interval; TNK = tenecteplase; ALT = alteplase.
